# Visuocortical tuning to a threat-related feature persists after extinction and consolidation of conditioned fear

**DOI:** 10.1038/s41598-020-60597-z

**Published:** 2020-03-03

**Authors:** Martin I. Antov, Elena Plog, Philipp Bierwirth, Andreas Keil, Ursula Stockhorst

**Affiliations:** 1 Institute of Psychology, Experimental Psychology II and Biological Psychology, University of Osnabrück, D-49074 Osnabrück, Germany; 2 Department of Psychology and Center for the Study of Emotion and Attention, University of Florida, Gainesville, Florida 32611 USA

**Keywords:** Attention, Consolidation, Extinction, Fear conditioning, Visual system

## Abstract

Neurons in the visual cortex sharpen their orientation tuning as humans learn aversive contingencies. A stimulus orientation (CS+) that reliably predicts an aversive noise (unconditioned stimulus: US) is selectively enhanced in lower-tier visual cortex, while similar unpaired orientations (CS−) are inhibited. Here, we examine in male volunteers how sharpened visual processing is affected by fear extinction learning (where no US is presented), and how fear and extinction memory undergo consolidation one day after the original learning episode. Using steady-state visually evoked potentials from electroencephalography in a fear generalization task, we found that extinction learning prompted rapid changes in orientation tuning: Both conditioned visuocortical and skin conductance responses to the CS+ were strongly reduced. Next-day re-testing (delayed recall) revealed a brief but precise return-of-tuning to the CS+ in visual cortex accompanied by a brief, more generalized return-of-fear in skin conductance. Explorative analyses also showed persistent tuning to the threat cue in higher visual areas, 24 h after successful extinction, outlasting peripheral responding. Together, experience-based changes in the sensitivity of visual neurons show response patterns consistent with memory consolidation and spontaneous recovery, the hallmarks of long-term neural plasticity.

## Introduction

Classical fear conditioning is a fundamental process of learning and memory in humans and other animals^1^. It enables them to predict threats from cues in the environment and to disregard cues that are no longer predictive. Fear conditioning is also a widely used model in basic neuroscience, psychiatry and neurology. During acquisition in the laboratory, a neutral stimulus predicting an aversive unconditioned stimulus (US) becomes conditioned (CS) and capable of eliciting a defensive response when presented alone. In extinction learning, the CS is repeatedly presented without the US and conditioned responses decline^1–3^. The neural circuitry of fear acquisition and extinction is well established in rodent and primate models and includes the “fear network” of the amygdala, hippocampus, and prefrontal cortex. However, both fear acquisition and extinction induce plastic changes in a wider brain network, including the brain’s sensory systems^4–6^. This is well documented in the primary and extended auditory cortex for rodents^7–13^ and in few neuroimaging studies in humans^14–17^. By contrast, much less is known about the role of the visual cortex in associative memory and fear conditioning^5^. In general, visual neurons show experience-dependent plasticity, outside the critical periods of development^5,18^. Furthermore, human visuocortical responses to the CS+ predicting a threat are amplified during *fear acquisition*^19–23^, resulting in a prioritized processing of threat-predictive cues. Simultaneously, responses to non-predictive CS− (safety cues) are inhibited^24,25^. This visual processing bias is linked to improved perceptual discrimination^26^. Importantly however, it is not clear if the changes in visual processing measured during the learning process are temporary or if they are part of a long-term memory trace.

Limited evidence suggests that cortical processing is responsive to quick changes in the predictive value of cues but also stable enough to support long-term memory. In *extinction learning*, presenting the visual CS alone can reverse associative changes in the visual^25,27^ and for auditory CS in the primary auditory cortices^5,7^. Yet, in the auditory association cortex, animal^28–30^ and human^31^ studies have converged to demonstrate fear-conditioned changes that persisted despite extinction learning. *Extinction* includes learning a new CS-no US association^32,33^, leaving the original CS-US memory trace mostly intact. At later recall, the competition between the CS-US and the CS-noUS memory traces decides if and how much conditioned responding is shown^32^. Extinguished responses are vulnerable to return-of-fear phenomena, including spontaneous recovery, where conditioned responses return with the mere passage of time^1,33,34^. Yet, the role of the visual cortex in long-term reduction (i.e., good extinction recall) vs. persistence of conditioned responding (i.e., spontaneous recovery) after extinction learning is not clear, hampering our understanding of the neurophysiological mechanisms mediating return-of-fear. Elucidating its role has strong implications regarding the aetiology of affective and anxiety disorders e.g., for understanding persistent attention biases to anxiety-related cues, or visually-driven flashbacks in the face of fear-related stimuli.

**Figure 1:**
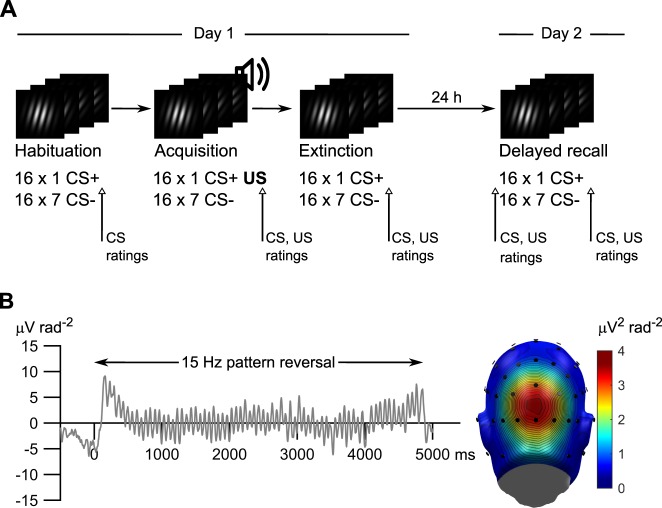
Experimental procedure and steady-state evoked potential (ssVEP) time domain and topographical distribution. (**A**) Overview of the procedure and example stimuli used during the 4 phases of the experiment: Habituation, acquisition, extinction, and a 24-h delayed recall. During each learning phase participants (*N* = 19) passively viewed high-contrast grating stimuli with eight different orientations (16 trials for each orientation and learning phase). To evoke a steady-state brain response with a known frequency, each grating reversed phase 71 times per trial at a rate of 15 (*N* = 9) or 14.167 Hz (*N* = 10). This produces the visual impression of the grating jumping slightly from left to right at a steady pace. Only during the acquisition phase one of the gratings (the CS+) was paired with a 1-s, 98 dB (**A**) aversive white noise burst (unconditioned stimulus = US). (**B**) Evoked steady state visuocortical response. *Left:* Representative time domain signal from the middle occipital sensor (Oz) for the 15 Hz phase reversal of one grating orientation (45°), averaged over 16 habituation trials and *N* = 9 participants. *Right:* Scalp distribution of the frequency domain average of the 15 Hz ssVEP power for the same stimulus and participants.

In the present study, we tested the alternative hypotheses that visuocortical responses will show extinction recall or spontaneous recovery after a consolidation period. Orientation selectivity is a fundamental organizational property of neurons in the lower-tier, especially the primary visual cortex (V1)^35,36^. Based on previous work^25^, we used steady-state visually evoked potentials (ssVEP) as a direct measure of sustained large-scale neural population activity, generated in lower-tier retinotopic cortex^37^, to investigate trial-by-trial changes in cortical orientation selectivity. In a generalization paradigm^25^, we used seven gratings, differing only in orientation (increasing linearly in 10° steps), as CS. Only one (45°, CS+) was paired with an aversive noise US during acquisition. Due to lateral inhibition of orientation columns in the visual cortex^38^, and based on previous findings^25^, we expected fear acquisition to amplify cortical responses to the CS+ but reduce them for the most similar CS−, resulting in a ‘Mexican hat’ tuning pattern. To allow for consolidation, we used a 2-day procedure (Fig. 1A) with acquisition and immediate extinction learning on day 1 and a 24-h delayed-recall test on day 2. To validate the extent to which learning took place, we assessed conditioned skin conductance responses (SCR, an indicator of sympathetic nervous system activity), subjective ratings of CS valence and arousal (evaluative learning), and collected US expectancy ratings (contingency knowledge). For those responses, we expected to see a gradual decrease in responding with decreasing similarity to the CS+, i.e. stimulus generalization^25,39,40^ instead of lateral inhibition.

## Results

### Increased visual cortex responding and selective amplification of the threat-predictive stimulus during fear acquisition

To examine if learned changes in the orientation tuning of visual cortex neurons represent a long-term CS-US memory trace, we recorded 64-channel electroencephalography (EEG) from 19 male volunteers in a 2-day fear conditioning task (Fig. 1A). On day 1, participants first underwent habituation. High-contrast gratings with 8 different orientations (15°, 25°, 35°, 45°, 55°, 65°, 75°, and control −45°, Fig. 1A) were presented 16 times each in a pseudorandom order. For acquisition, the grating CS were presented again 16 times, the 45° grating (the CS+) co-terminating with a 1-s aversive 98 dB (A) white-noise burst (the US). Extinction learning consisted of 16 presentations of each CS, without the US. After 24 h, delayed recall on day 2 was identical to the extinction phase. We delivered the grating CS in a phase-reversing stream, where the presentation alternated between the phase and counter phase version of a grating at a rate of 14.167 or 15 Hz (7.09 & 7.5 Hz for a full cycle). This evoked a robust phase-reversal ssVEPs at the second harmonic of the full cycle frequency, i.e., at 14.167 and 15 Hz, respectively, with a peak over the occipital pole (Fig. 1B).

**Figure 2:**
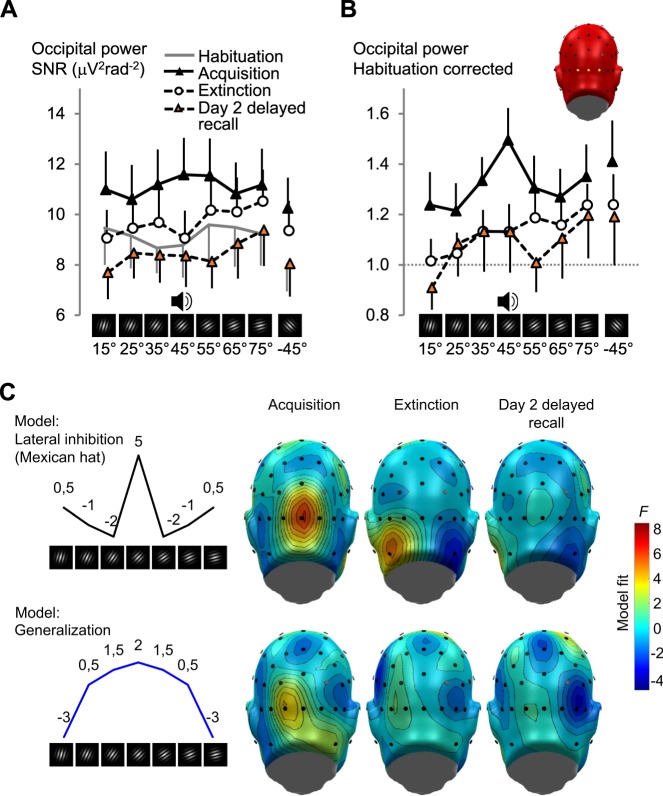
Occipital cortical responses during the different phases of conditioning. (**A**) Changes in the grand average (*N* = 19) of visual electrocortical activity for each learning phase (habituation, acquisition, extinction, and day 2 delayed recall) and for each CS orientation. Regional means of the ssVEP spectral power current source density (CSD, Laplacian space), averaged across 3 occipital midline sensor locations (O1, Oz, O2), were used to estimate the occipital cortex surface potential. Values are signal-to-noise ratios (SNR), i.e., the power at the driving frequency was divided by the average power for the five frequency bins below and four frequency bins above the driving frequency (as the noise estimate). Supplementary Fig. S1 shows single subject data. (**B**) The same data after habituation correction for acquisition, extinction, and day 2 delayed recall. The insert shows a view of the back of the electrode array used, the sensor locations used for averaging are highlighted. Error bars show 1 standard error of the mean (*SEM*). Supplementary Fig. S2 shows single subject data. (**C**) Cortical regions responsive to fear conditioning: Topographical distributions (back views of the scalp) showing results (*F*-values with *N* = 19) of planned contrasts testing for lateral inhibition (top, black line, ‘Mexican hat’ contrast) versus fear generalization (bottom, blue line, quadratic contrast) of habituation-corrected electrocortical responses across orientations, averaged over all acquisition, extinction, and day 2 delayed recall trials. *F*-values exceeding ±4.41 indicate a reliable model fit. Fits matching the opposite pattern (i.e., inverted ‘Mexican hat’ or quadratic) are shown in blue. The numbers above the line-graphs on the left are the weights used for planned contrasts.

We explored how occipital cortex activity is shaped by fear acquisition and extinction across the two experimental days. For this, we first pooled occipital activity from current source density (CSD) spectral power at the driving frequency for three a priori defined sensors around the occipital pole (Oz, O1, and O2, Fig. 1B), where we expected the hypothesized learning effects^25^. Occipital activity was subjected to repeated-measures ANOVA (learning phase [4] × CS orientation [8]) and planned contrasts, designed to test the competing hypotheses of *lateral inhibition* (modelled as a ‘Mexican hat’ function) vs. fear *generalization* (modelled as a quadratic function, Fig. 2C shows the weights). We used a Bayesian information criterion (BIC)^41^ to formally compare the models and report the difference (ΔBIC), where values >2, suggest *positive*, >6 *strong*, >10 suggest *very strong* evidence to prefer the hypothesized model over the alternative^42,43^.

We found a significant effect of learning phase (*F*_(1.7,30.2)_ = 4.756, *p* = 0.021, part. η^2^ = 0.209, *N* = 19), due to higher occipital ssVEP power during acquisition **(**Fig. 2A). The effect was not confined to the CS+ (CS orientation × Learning phase interaction: *F* < 1; CS orientation: *F*_(3.0,54.0)_ = 2.300, *p* = 0.088, part. η^2^ = 0.113; contrasts for acquisition, both *F* < 1).

We repeated the analysis after a habituation correction (Fig. 2B), normalizing each subject’s occipital power for each CS through division by the corresponding orientation’s mean habituation power – yielding a relative ssVEP change index. Planned contrasts on the habituation corrected data showed a significant ‘Mexican hat’ fit (*F*_(1,18)_ = 4.806, *p* = 0.042, *r*^2^_contrast_ = 0.202) during acquisition **(**Fig. 2B), but not in extinction or day 2 delayed recall (both *F* < 1). The competing generalization model did not show a significant fit to data from acquisition (*F*_(1,18)_ = 0.66, ΔBIC_(G-L)_ = 3.6, favouring lateral inhibition), extinction or day 2 delayed recall (both *F* < 1). Figure 2C shows the topographical distribution of the lateral inhibition (top row) vs. generalization (bottom row) model fits across the posterior part of the sensors (back view) for habituation-corrected data averaged across all trials of the learning phases acquisition, extinction, and day 2 delayed recall. In sum: besides a general increase in occipital cortex responding during acquisition, correcting for habituation revealed a selective enhancement of occipital ssVEP power for the threat-predictive CS+. Supplementary Figs. S1 and S2 show single subject data.

### Single trial analysis reveals fast extinction on day 1 and a brief return-of-tuning in the occipital cortex

**Figure 3:**
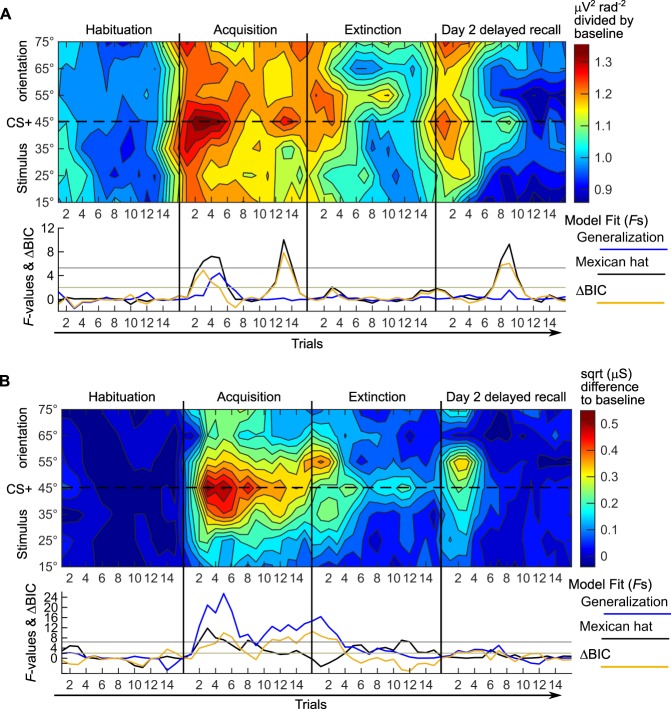
Trial-by-trial development of occipital orientation tuning and sympathetic skin conductance responses (SCR) during conditioning over two days. (**A**) Single-trial cortical responses, pooled across 3 occipital midline sensor locations (O1, Oz, O2). *Top panel:* color-coded single-trial amplitude of the occipital visual electrocortical response during habituation, acquisition, extinction, and day 2 delayed recall. The dynamic of learning and recall in the visual cortex is shown for the 8 CS orientations (shown on the y-axis) with the CS+ (45 degree orientation) in the middle. *Bottom:* model fits (*F*-values from planned contrasts) for the competing hypotheses of fear generalization (blue line) and lateral inhibition (black line, ‘Mexican hat’), calculated for each trial. The yellow line shows the ΔBIC_(G-L)_ = BIC(Generalization) − BIC(Lateral inhibition), values >2 favour lateral inhibition, values <−2 favour generalization. Note: Contour plots show no error estimates, see Supplementary Fig. S5 for an alternative depiction. (**B**) Single-trial SCR. *Top:* As in (**A**) but for average color-coded single-trial amplitude of the SCRs. *Bottom:* model fits (planned contrasts) for fear generalization (blue line) and lateral inhibition (black line). The yellow line shows the ΔBIC_(L-G)_, as we expected generalization for SCR, here values >2 favour generalization, values <−2 favour lateral inhibition. In both panels: where the data fit an inverted quadratic or ‘Mexican hat’ contrast, the *F*-values were given a negative sign to denote the inverted fit.

Learning is typically a time-dynamic process and the quality of its outcome changes as a function of the number of training trials. Therefore, we utilized the excellent signal-to-nose ratio of steady-state visually evoked potentials (ssVEPs) to capture trial-by-trial changes in the orientation sensitivity of visual cortex mass neural activity. After converting the ssVEP to current source density (CSD) estimates of cortical surface power at the driving frequency, we pooled the trial-by-trial power averaged for the 3 a priori defined sensors nearest to the occipital pole (Oz, O1, O2, Fig. 3A). For each orientation, single-trial power was normalized by dividing by the habituation mean for the same orientation (all 16 trials). The critical *F*-values for the competing lateral inhibition (‘Mexican hat’) vs. generalization (quadratic) models were obtained by calculating permutation distributions on data shuffled across orientations within each participant (4000 permutations), yielding a critical *F*-value of 5.282. The associated ΔBIC_(G-L)_ (generalization – lateral inhibition), are shown by the yellow line in (Fig. 3A).

As previously reported^25^, a tuning to the 45° grating signalling the aversive loud noise burst (CS+) emerged quickly. After only two trials during the acquisition phase (Fig. 3A), the third, fourth, and fifth trial showed a significant ‘Mexican hat’ fit. However, our data also showed that the tuning to the CS+ fluctuated over trials: After the initial tuning, there was a brief reduction. The CS+ tuning then re-emerged again stronger at the end of acquisition (trials 13 and 14). This fluctuation of tuning over trials might explain the lack of statistical significance when subjecting ssVEP averaged over all trials of the learning phase to an ANOVA. During extinction learning trials (day 1), the tuning disappeared almost instantly and overall activity was reduced (Fig. 3A). On day 2, there was a diffuse increase in activity during the initial 3 trials of delayed recall that did not result in a significant generalization or lateral inhibition fit. Interestingly, a tuning to the CS+ re-emerged after 24 hours during delayed recall, most pronounced during trials 8, 9, and 10. Here, activity to the CS+ was enhanced while activity to the 2 most similar CS− was strongly suppressed (Fig. 3A). This resembles a memory consolidation effect and return-of-tuning, despite extinction on day 1 at the level of mass sensory cortical activity. Supplementary Fig. S5 shows the data as conventional line plots with error bars.

### Trial-by-trial changes in skin conductance responses show a similar temporal dynamic as occipital cortex responses

In order to describe the temporal dynamics of the sympathetic conditioned responding, we also conducted a single-trial analysis for skin conductance responses. Similar to single-trial ssVEP analysis, we first corrected for habituation level by subtracting the average over 16 habituation trials from each response within a participant and orientation. The result is plotted in Fig. 3B. Again, we fitted *F*-contrasts representing the lateral inhibition (‘Mexican hat’) and generalization (quadratic) response patterns. The critical *F*-value (obtained by calculating permutation distributions with 4000 permutations) was 6.396. For acquisition, a quickly emerging and prominent generalization pattern (Fig. 3B) was the best fit for the data.

As with the lateral inhibition pattern in single-trial ssVEP, the SCR-generalization pattern fluctuated over trials. There was strong generalization for acquisition trials 2–6, followed by a reduction for trials 8–10, and a re-emergence of generalized responses towards the last trials of fear acquisition. Generalization subsided within the first four trials of extinction. During day 2 delayed recall, the single-trial SCR showed a diffuse return-of-fear confined to the first two trials and with a maximum for the 55° CS, followed by the 45° CS+. This pattern did not reach significance in either of the two contrasts (both centred on the 45° CS+).

### Lateral temporo-occipital cortex shows persistent tuning to the CS+ 24 h after extinction

**Figure 4:**
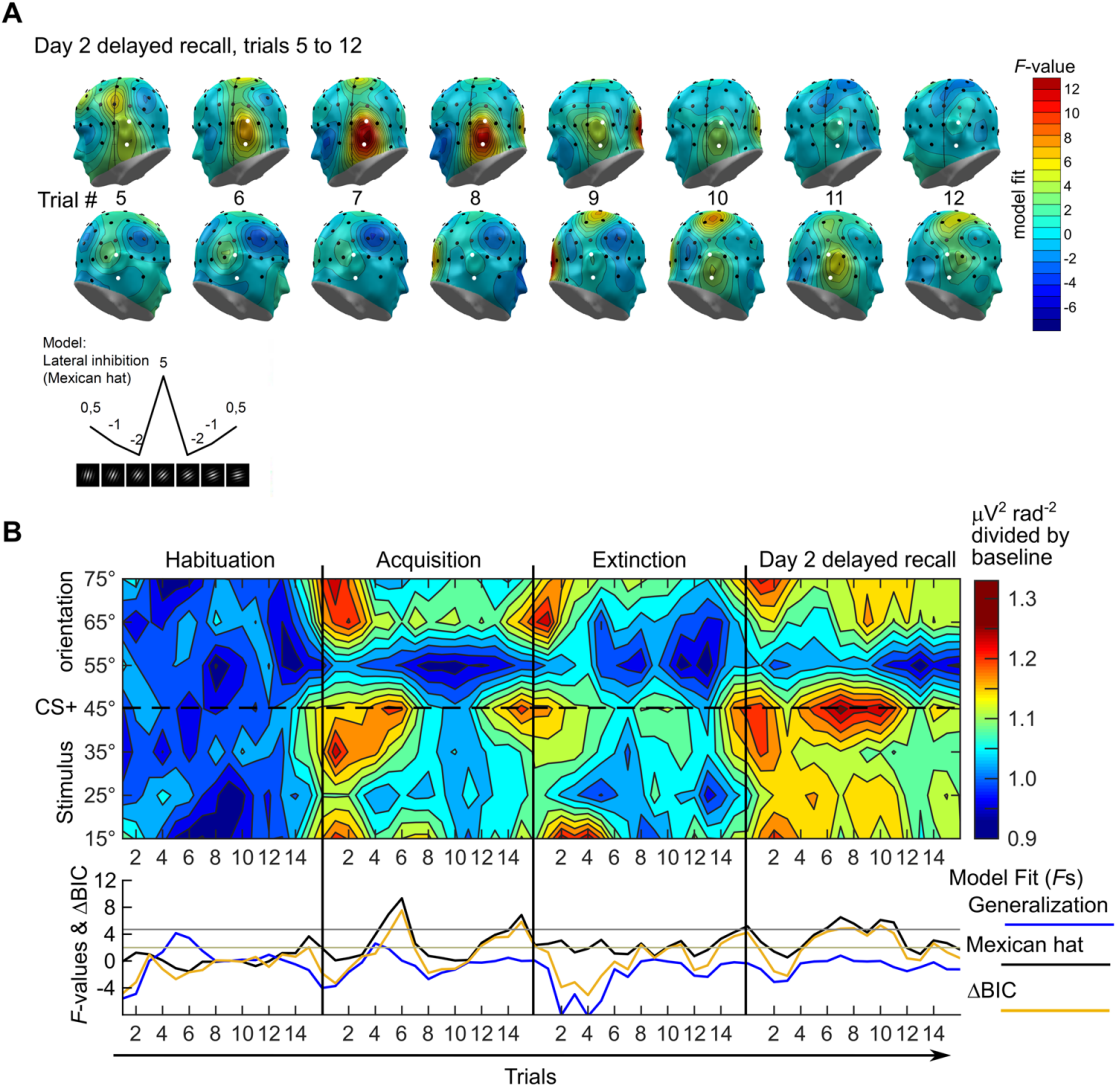
Conditioned tuning over lateral temporo-occipital cortex in single-trial data. (**A**) Topographical distributions (left and right views of the scalp) showing results (*F*-values) of contrasts testing for lateral inhibition (‘Mexican hat’) for habituation-corrected electrocortical responses across orientations, for trials 5–12 of day 2 delayed recall. The 4 sensors selected for analyses are highlighted white. Fits matching the opposite pattern (i.e., inverted ‘Mexican hat’) are shown in blue. (**B**) Trial-by-trial development of orientation tuning during conditioning. Top: color-coded single-trial amplitude of the exploratory 4-sensor cluster from the bilateral temporo-occipital region (TP8, TP10, TP7, and TP9), shown for the 7 CS orientations (on the y-axis) with the CS+ (45° orientation) in the middle. *Bottom:* model fits (planned contrasts, *F*-values) for generalization (blue line) and lateral inhibition (black line, ‘Mexican hat’), calculated for each trial. The yellow line shows the ΔBIC_(G-L)_ = BIC(Generalization) − BIC(Lateral inhibition), values >2 favour lateral inhibition, values <−2 favour generalization. Where the data fit an inverted quadratic or ‘Mexican hat’ function, the *F*-values were given a negative sign to denote the inverted fit. Note: Contour plots show no error estimates, see Supplementary Fig. S5 for an alternative depiction. For completeness, Supplementary Fig. S9 shows 16-trial averages of these 4 sensors, as in Fig. 2A,B.

Cortical responses after extinction learning and a consolidation period were not examined until now. Therefore, in an explorative analysis we looked for regions showing prolonged tuning to the CS+ on day 2 over all sensor locations. An examination of the topographical distribution (habituation-corrected data, averaged across all trials per learning phase) revealed a bilateral region showing a ‘Mexican-hat’ fit for acquisition, extinction, and (more lateralized to the left) also during delayed recall on day 2 (data not shown). Inspection of single-trial data however, showed a similar but more posterior location including sensors TP7 and TP9 on the left, and to a smaller amount also for the corresponding sensors on the right (TP8, TP10). Figure 4A shows a scalp topography of this effect for trials 5–12 of the delayed recall on day 2, where the fit was most pronounced. Supplementary Figs. S3 and S4 show this for all trials and every learning phase. Therefore, we repeated the single-trial analysis for this bilateral temporo-occipital region pooled from the explorative 4-sensor cluster (TP8, TP10, TP7, and TP9). The critical *F*-value for this 4-sensor cluster (again determined through permutation testing with 4000 iterations) was *F* = 4.70. The results (Fig. 4B) show that for this cluster there was a prolonged and more persistent tuning to the CS+, following a ‘Mexican hat’ pattern and lasting throughout 11 of the 16 trials of day 2, despite the lack of significant tuning during extinction learning on day 1 (Fig. 4B). Supplementary Fig. S5 shows the data as conventional line plots with error bars.

### Sympathetic arousal and subjective responses to the CS validate successful learning and show fear generalization

**Figure 5:**
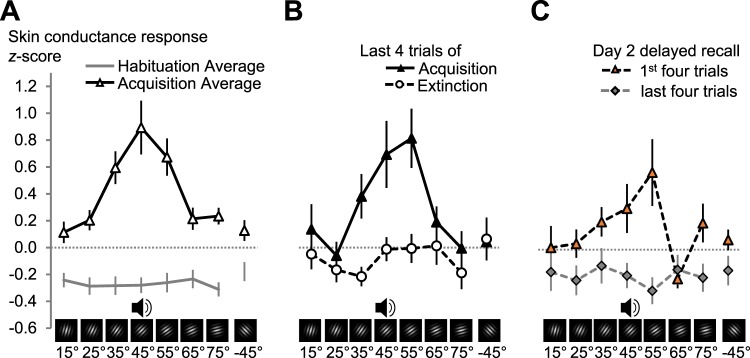
Skin conductance responses during the different phases of fear conditioning. (**A**) Mean (*N* = 19) skin conductance responses averaged over all 16 trials of habituation and acquisition on day 1. (**B**) Shows the means for the last four trials of acquisition and extinction on day 1, respectively. (**C**) The first and last four trials of the delayed recall phase on day 2. In all plots the data are averaged over individual z-scores, standardized on the mean and *SD* of all CS responses of a subject in the experiment. Error bars show ± 1 *SEM*. Supplementary Fig. S6 shows single subject SCR data.

Finally, to further validate our task with more typical measures of human fear learning, we analysed participant’s averaged skin conductance responses (SCR), subjective ratings of CS valence, CS arousal, and US expectancy. As expected, SCR, as an indicator of sympathetic arousal, showed successful learning. While the CS orientation had no effect on SCR during habituation (*F* < 1), there was a significant impact of CS orientation during acquisition (Fig. 5A, *F*_(2.7,49)_ = 7.966, *p* = 0.0003, part. η^2^ = 0.307; CS orientation × Learning phase interaction: *F*_(3.3,60)_ = 8.396, *p* = 0.00006, part. η^2^ = 0.318). During the acquisition phase, SCR followed the expected conditioned generalization gradient, favouring the CS+ and the two most similar CS−. Indeed, SCR during acquisition were best modelled by a quadratic function with the highest responses to the CS+ (Table 1 summarizes the results from the planned contrasts, and model comparisons for generalization and lateral inhibition for SCR and subjective ratings).

**Table 1:** Results of planned contrasts for generalization and lateral inhibition and model comparison for SCR, subjective CS arousal ratings, and US expectancy ratings.

SCR (*N* = 19)	Generalization (quadratic)			Lateral inhibition (‘Mexican hat’)			
	*F*_(1,19)_	*p*	*r*^2^_contrast_	*F*_(1,19)_	*p*	*r*^2^_contrast_	ΔBIC_(L-G)_
Habituation (16 trials)	0.01	0.910	0.001	−0.08	0.776	0.004	−0.1
Acquisition (16 trials)	27.81	0.0001	0.594	7.37	0.014	0.280	10.9
Acquisition last 4 trials	11.61	0.003	0.379	1.12	0.304	0.056	8.0
Extinction last 4 trials	0.34	0.567	0.018	0.60	0.449	0.031	−0.3
Day 2 first 4 trials	2.37	0.141	0.111	0.10	0.757	0.005	2.1
**Arousal ratings (*N*** = **19)**							
Habituation	−0.10	0.753	0.073	−0.47	0.500	0.156	−0.4
Acquisition	20.19	0.0003	0.718	9.12	0.007	0.569	6.3
Extinction	23.85	0.0001	0.746	1.59	0.223	0.278	13.9
Day 2 (before)	16.81	0.001	0.685	4.55	0.047	0.440	8.0
Day 2 (after)	9.44	0.007	0.576	6.30	0.022	0.499	2.2
**US expectancy (*N*** = **18)**							
Acquisition	28.32	0.0001	0.782	13.08	0.002	0.649	7.2
Extinction	18.34	0.001	0.710	2.10	0.165	0.323	10.7
Day 2 (before)	26.04	0.0001	0.769	11.59	0.003	0.626	7.2
Day 2 (after)	12.95	0.002	0.647	7.98	0.012	0.554	3.1

*Note*. ΔBIC_(L-G)_ is the difference of the estimated Bayesian information criteria for the two competing models. We expected to see generalization for SCR, CS arousal ratings, and US expectancy ratings. Therefore, here ΔBIC_(L-G)_ = BIC_(Lateral inhibition)_ − BIC_(Generalization)_, where ΔBIC >2, suggests positive, ΔBIC >6 suggests strong, and ΔBIC >10 suggest very strong evidence to prefer the generalization model over lateral inhibition. US expectancy has *N* = 18 due to missing ratings from one participant, the associated *F-*values have 1 and 18 degrees of freedom.

Extinction learning markedly reduced SCR-responding (Fig. 5B, Learning phase [last 4 acquisition trials vs. last 4 extinction trials]: *F*_(1,18)_ = 8.246, *p* = 0.010, part. η^2^ = 0.314), due to a drop in responding to the CS+ and perceptually similar CS− (CS orientation × Learning phase: *F*_(7,126)_ = 2.644, *p* = 0.014, part. η^2^ = 0.128). During the last 4 extinction trials there was no effect of CS orientation (*F*_(7,126)_ < 1.11, Fig. 5B) or evidence of generalization around the CS+ (Table 1). Despite the successful reduction with extinction learning on day 1, the conditioned effect of CS orientation returned after 24 h of consolidation during the first 4 trials on day 2 (last 4 extinction trials vs. first trials on day 2: Fig. 5C, CS orientation × Learning phase: *F*_(7,126)_ = 2.680, *p* = 0.013, part. η^2^ = 0.130, CS orientation main effect for the first 4 trials on day 2: *F*_(3,53.3)_ = 3.259, *p* = 0.029, part. η^2^ = 0.153). However, the largest responses during the first 4 trials on day 2 were for the 55° grating, not for the 45° CS+ and the quadratic fit failed to reach significance (Table 1).

**Figure 6:**
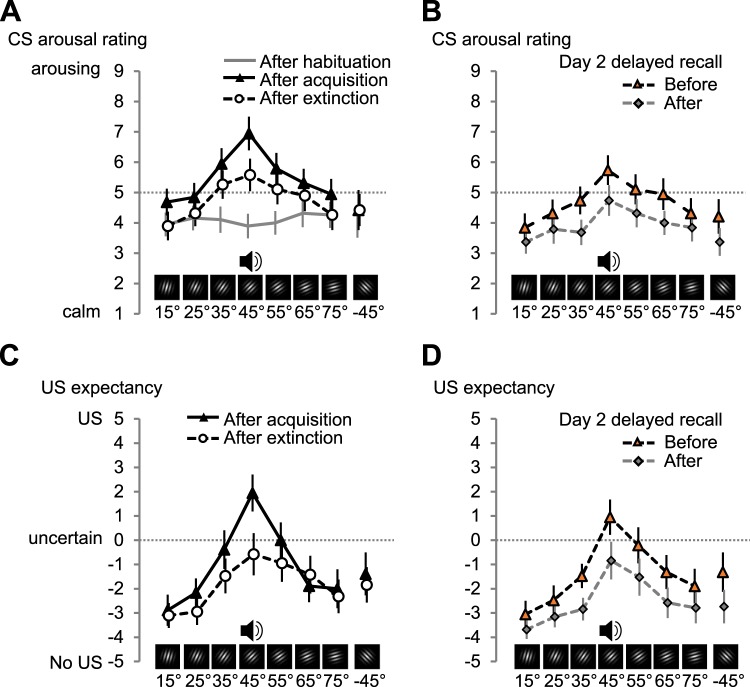
Subjective self-report changes in CS arousal and US expectancy during the different phases of fear conditioning. Mean (*N* = 19) arousal rating for each CS orientation as rated (**A**) after habituation, acquisition, and extinction on day 1 and (**B**) before and after delayed recall on day 2. US expectancy mean (*N* = 18) as rated (**C**) after habituation, acquisition, and extinction on day 1 and (**D**) before and after delayed recall on day 2. Error bars show ±1 *SEM*. Supplementary Figs. S7 and S8 show single subject data for arousal and US expectancy ratings.

For *subjective responses to the CS*, we first compared the arousal and valence ratings collected after habituation, acquisition, and extinction learning on day 1. When rated after habituation, all CS where evaluated as relatively calm (CS main effect: *F* < 1, Fig. 6A). This changed after acquisition (CS × Time point: *F*_(4,71.1)_ = 4.080, *p* = 0.005, part. η^2^ = 0.185). Now the CS+ (45°) and its two nearest neighbours were rated as more arousing (CS effect: *F*_(2.9,52.8)_ = 4.361, *p* = 0.009, part. η^2^ = 0.195), following a generalization gradient (Table 1). After extinction, the arousal ascribed to the stimuli was reduced (Time point: *F*_(1,18)_ = 7.655, *p* = 0.013, part. η^2^ = 0.298) but an enhancement for the CS+ and its neighbours remained (CS: *F*_(2.3,42.1)_ = 3.906, *p* = 0.022, part. η^2^ = 0.178), best modelled by generalization (Table 1). This indicates a reduction, but not a complete extinction of generalized subjective arousal. On day 2 (Fig. 6B), arousal ratings collected before delayed recall were comparable to those measured after extinction on day 1 (Time point and CS × Time point, both *F* < 1, CS: *F*_(2.5,45.8)_ = 3.471, *p* = 0.030, part. η^2^ = 0.162, Table 1). Even after day-2 extinction trials, there was a remaining CS effect (Fig. 6B, *F*_(3.4,60.4)_ = 3.192, *p* = 0.025, part. η^2^ = 0.151), again modelled better by generalization around the CS+.

For valence ratings we found similar results (data not shown). Again, compared to habituation, the rating of the CS+ and its nearest neighbours became more negative after acquisition (CS × Time point: *F*_(2.9,51.7)_ = 4.119, *p* = 0.012, part. η^2^ = 0.186), and this was not abolished after extinction learning on day 1 (CS × Time point: *F* < 1, CS effect: *F*_(2,35.3)_ = 3.165, *p* = 0.055, part. η^2^ = 0.150). In contrast to arousal ratings, conditioned changes in valence were not significant on day 2, neither before nor after the 16 extinction trials on day 2 (CS effects: *F*_(2,35.8)_ = 1.705, *p* = 0.196, part. η^2^ = 0.087, and *F*_(2.6,46)_ = 1.682, *p* = 0.190, part. η^2^ = 0.085, respectively).

We also asked participants to rate for each CS, to what extent they expected it to be followed by the loud noise US (−5 being 100% certain that *no* US would follow, 0 being uncertain, +5 being 100% certain that *this CS would be followed* by a US). We refrained from a US expectancy rating after habituation because it could introduce anticipation effects during acquisition. After acquisition participants were able to identify the CS+ (Fig. 6C, CS effect: *F*_(3.7,59)_ = 6.859, *p* = 0.0002, part. η^2^ = 0.300). Consistent with generalization (Table 1), the US expectancy was highest for the CS+, with the neighbouring two gratings rated as close to uncertain and the more dissimilar gratings rated as relatively safe (Fig. 6C). After extinction, the US expectancy was reduced, especially for the CS+ (CS × Time point: *F*_(4,64)_ = 2.651, *p* = 0.041, part. η^2^ = 0.142), with remaining bias towards the CS+ (CS: *F*_(2.7,47.9)_ = 3.583, *p* = 0.024, part. η^2^ = 0.166), modelled by generalization (Table 1). On day 2 (Fig. 6D), before delayed recall the US expectancy was only descriptively higher (vs. end of day 1, CS × Time point: *F*_(4.4,79.4)_ = 1.320, *p* = 0.268, part. η^2^ = 0.068, CS: *F*_(2.7,47.7)_ = 5.590, *p* = 0.003, part. η^2^ = 0.237). The generalization pattern was attenuated, but not abolished at the end of day 2 (main effect CS: *F*_(3.1,56.3)_ = 3.659, *p* = 0.016, part. η^2^ = 0.169, Table 1).

## Discussion

Prior work^25^ has established that pairing a specific orientation with an aversive outcome changes orientation selectivity of neuronal populations in the human visual cortex, accompanying the learning of behavioural relevance. We examined if this acquired change in orientation selectivity persists after extinction learning and a subsequent consolidation interval. As expected, conditioning prompted defensive mobilization at the level of autonomic arousal, biased CS evaluation, and produced explicit contingency knowledge, reflected in increased SCR, subjective ratings of arousal, aversion, and US expectancy. In these four measures responding decreased gradually with increasing angular distance from the CS+, showing a pronounced generalization that decreased but did not disappear with extinction learning. In contrast, visual cortex responding during acquisition was selectively amplified for the CS+ while the most proximal, but not distal, orientations were suppressed, thus following a ‘Mexican-hat’ function^25^. Such tuning has also been observed in studies of adaptation and feature-based selective attention, where it is often reflected in behavioural and neural population variables^25,36^. It is not known if the origin of emerging lateral inhibitory patterns varies with the task, or if it represents a mechanism common to learning, adaptation, and instructed attention. Future research is needed to examine the differences and similarities between visuocortical prioritization mechanisms under different behavioural constraints. The present study contributes to this emerging literature by providing information regarding the longer-term sequelae of adaptive changes in population tuning: The tuning to the CS+ quickly subsided with immediate extinction on day 1. Despite this, and without further pairing of the CS+ with the US, a strong tuning to the CS+ reappeared briefly on day 2. The trial-by-trial timing of occipital tuning over the course of the two experimental days resembled the temporal progression of conditioned peripheral arousal responses, indexed by SCR. Rather than lateral inhibition, single-trial SCR displayed generalization, with a fast extinction on day 1 and brief return of responding on day 2. Explorative single-trial analyses revealed a temporo-occipital area where cortical tuning to the CS+ on day 2 was present throughout the majority of unreinforced day 2 trials. Our results show that biased visual cortex processing can persist after extinction training and 24 h of consolidation, while remaining flexible enough to reflect short-term changes in the predictive relevance of stimuli. The latter is supported by the rapid reduction in cortical responses during extinction learning in our data. Recent single-trial MEG data show changes in the retinotopic visual cortex within only a few trials of reinforced or unreinforced CS^44^.

Unlike previous studies^25^, we found that occipital responding to all CS orientations increased during fear acquisition compared to habituation (Figs. 2A and 3A). This was in addition to responses following a ‘Mexican hat’ pattern, tuned to the CS+, as reported previously^25^. Together, this could indicate two different mechanisms coexisting during acquisition: one increasing overall cortical gain, the second tuning sensitized responding in accordance with the behavioural relevance of stimuli to enable discriminability. In behavioural terms, these mechanisms may be related to enhanced vigilance and sensory discrimination, respectively. Broader increases in cortical gain may reflect overall arousal associated with noradrenergic signalling^45^.

Importantly, we also show a return of cortical orientation tuning on day 2, despite its abolishment in day-1 immediate extinction. This supports two interpretations: (1) Changes in cortical responding acquired during fear conditioning seem to be subject to consolidation processes over the 24-h retention period – a hallmark of plasticity and a clue for the formation of a long-term memory; and (2) The learned changes in cortical orientation tuning follow the rules of associative, rather than perceptual learning. Return of extinguished cortical tuning to the CS+ is consistent with spontaneous recovery, a known phenomenon in conditioning^1,34^. This cannot be explained by processes of perceptual learning, in which the mere exposure to the stimuli would result in plasticity. The explorative finding of persistent tuning to the CS+ in bilateral temporo-occipital cortical areas, despite day 1 extinction and additional extinction on day 2, is of special interest. It may signify a process resembling systems consolidation: cortical processing on day 2 may recruit a different set of visual neurons after the 24-h consolidation period. Indeed, findings from auditory conditioning suggest that older memories may rely more on the higher auditory cortex than newly acquired ones^46,47^.

The return of visual cortical tuning observed in the present study may contribute to our understanding of the role of the visual cortex in fear extinction and more generally adds to an ongoing debate about the nature of fear extinction. Return-of-fear phenomena are reliably shown at the behavioural level. Therefore, most contemporary accounts consider extinction as comprising learning a new inhibitory CS-noUS association^2,48,49^. However, at the level of changes in neural circuitry, findings support both – new learning and unlearning, depending on the neural system investigated^50–52^. Studies in mice show that fear acquisition with an auditory CS induces dendritic spine formation in the auditory cortex^53,54^. Yet, these newly formed spines were eliminated with extinction learning (2 days with 5 CS-alone trials per day)^53^, supporting the idea that the fear memory trace can be erased with extinction in the primary auditory cortex. Until now this has not been studied for fear-conditioned changes in the visual cortex. Our results show that the visual cortex is able to accomplish fast adaptation to changes in contingency (as seen in immediate extinction), while still exhibiting a long-term bias towards a stimulus that has a history of being dangerous (as evident in the return of cortical tuning on day 2).

Cortical areas showing prolonged or extinction-resistant responding to a fear conditioned stimulus may hold clinical relevance. They could be related to return-of-fear phenomena contributing to relapse after therapy in psychiatric disorders^55^, especially for visually-driven flashbacks. For example, increased detection of combat-related words in a rapid visual stream is related to increased visual cortex responding in combat-exposed soldiers with posttraumatic stress disorder compared to combat-exposed soldiers without PTSD^56^. In our data, visuocortical re-tuning, lasting several trials beyond the peripheral arousal responses on day 2, illustrates that visual cortical activity follows a different time course than typically used conditioning measures. Despite behavioural evidence for the safety of the CS+, acquired through extinction learning, such persistent changes may serve to prepare for a fast re-learning if the contingency changes again in the future. In auditory fear conditioning, similar prolonged and apparently extinction-resistant changes were found in the human higher-order auditory association cortex^31^, while activation of the primary auditory cortex during extinction learning predicted extinction success^57^.

A possible limitation of our findings is that the single extinction session we applied might not be sufficiently extensive to produce a stable reversal of plastic changes. A recent rodent study found that a single extinction session leads to new learning while multiple extinction sessions lead to erasure of the original fear memory in lateral amygdala neurons^58^. We also employed immediate extinction (i.e., temporally very close to fear acquisition), and some findings suggest that this is associated with more return-of-fear compared to extinction on a different day^59^. Future studies may address these alternative explanations. We also used ‘booster trials’ at the beginning of acquisition (5 of the first 8 trials were reinforced CS+ trials), therefore effects like repetition priming and other trial-order effects cannot be ruled out in our acquisition data. However, a very similar tuning pattern during acquisition has been observed in other work in the absence of booster trials^25^, and effects of trial-order during acquisition cannot account for the returning of tuning after 24 hours – the main finding of the present study. Finally, further studies should replicate and also extend the finding by using other (but the 45°) grating orientations as the CS+.

Our results cannot address the question what information is represented in visual cortex as learning progresses. Our method also does not allow us to pinpoint if any neuronal plasticity happens in the visual cortex itself. Changes in cortical processing measured with our EEG method may also be the result of re-entrant projections from upstream cortical areas exerting a top-down biasing signal on the visual cortex^60^. Nevertheless, animal studies have shown that visual neurons are able to encode different learning-specific information: For example, neurons in the rat V1 encoded the timing of a delayed reward^61^. A recent study^62^ identified two distinct populations of neurons in the mouse lateral visual association cortex (LVAC) during an appetitive food-reward learning task. One subset was sensitive to the orientation of a grating, irrespective of the outcome (palatable food, bitter liquid, no outcome). A second subset of neurons was sensitive to the predicted outcome (e.g. food) and motivational strength (i.e., satiety reduced tuning to a food predictive cue) and flexibly shifted their tuning curves with contingency reversal. Neurons with similar properties (coding predicted outcome, sensitive to motivation changes) were also found in the mouse V1^62^. Finally, these visual neurons were also sensitive to trial-by-trial reward history, i.e. responses to a food cue in a given trial would change depending on how many times this cue was rewarded in the preceding trials^62^. The latter corresponds well with human MEG findings^44^.

In summary, our findings demonstrate that the lower tier visual cortex responding not only shows flexible and fast adaption in response to changing contingencies associated with visual stimuli. Persistence of biased cortical processing after extinction and a consolidation interval is consistent with the notion that sensory systems participate in the distributed network encoding long-term fear memory^4,5,63^. More mechanistic animal studies are needed to investigate this hypothesis. Future studies may also aim to pinpoint the exact features of behaviourally relevant information represented in visual cortex activity: for example, arousal, prediction error signal, reinforcement timing, motivational strength, and reward history. As fear acquisition and fear extinction are both relevant models for psychiatric disorders related to trauma and stress, it will be important to establish the extent to which cortical processing bias towards danger signals is affected by additional experimental stress induction in humans.

## Materials and Methods

### Participants

Volunteers from the University of Osnabrück were screened for exclusion and inclusion criteria. Data from *N* = 19 male students between 18 and 29 years (*M* = 23.6, *SEM* = 0.68), with a BMI between 19.15 and 31.56 kg/m^2^ (*M* = 24.10, *SEM* = 0.43) comprised the final sample. Fear extinction and delayed recall are prone to sex differences linked to the female sex hormone 17-beta-estradiol^64,65^. Here, the sex-hormone question was not relevant. We therefore examined only men to avoid potentially confounding sex differences and fluctuations of sex-hormones in women. Participants were excluded in case of acute or chronic physical and psychiatric disorders (e.g., migraine, epilepsy, cardiovascular diseases, and phobias); also hearing impairments or tinnitus, left-handedness, uncorrected vision impairment, alcohol consumption of more than 40 g ethanol/day, drug abuse, smoking more than 5 cigarettes a day or any current medication. All participants were screened for posttraumatic stress disorder (PTSD) using a German version of the Posttraumatic Stress Diagnostic Scale^66^ and excluded if they met DSM-IV criteria for PTSD. From the 39 screened students, 9 did not meet the inclusion criteria, 4 additional did not appear for testing, and thus 26 subjects were assigned to the experiment. From those, 5 discontinued the ongoing experiment due to the aversive nature of the task, and one participant had to be excluded from analysis due to experimenter error. Finally, one participant was excluded from analysis because he failed to show an ssVEP signal over occipital areas even in a grand average over all trials (after visual inspection, and circular T-square statistic). The sample size was determined based on previous electroencephalography/magnetoencephalography fear conditioning studies^21,25,44,67^ with samples ranging from *N* = 15 to 21. We also considered sample sizes common in perceptual^26^, pharmacological^68^, and functional brain imaging studies^69^ of human fear conditioning and return- of-fear phenomena (*N* = 16 to *N* = 20, per group). The study protocol with all procedures and methods was approved by the ethics committee of the University of Osnabrück and was conducted in accordance with the Declaration of Helsinki guidelines. All procedures were carried out with the adequate understanding and written informed consent of the participants.

### Experimental design, fear conditioning stimuli and procedure

We used a differential fear conditioning protocol comprising four learning phases: habituation, acquisition and immediate extinction learning on day 1, and a 24 h-delayed recall on day 2. To examine if learned changes in the orientation tuning of visual cortex neurons represent a long-term CS-US memory trace, we recorded 64-channel EEG from our participants in this 2-day fear conditioning task (Fig. 1A). The stimuli, the basic learning task, and parts of the EEG analysis strategy follow earlier published work^25^.

The CS were high-contrast (maximum Michelson-Contrast: 96%) Gabor patches (sinusoidal gratings, filtered with a Gaussian envelope) with spatial frequency of 0.98 cycles/degree. They were presented centrally on a dark-grey background (100% black setting of the monitor) via a 19” cathode ray tube (CRT) monitor (visual angle 5.7°, vertically and horizontally). Participants viewed the CS while sitting in a comfortable chair in the electromagnetically shielded and sound attenuated experimental chamber, which was lit only by the CS presentation on the monitor during the conditioning phases. The CS had 8 different orientations with angles of 15°, 25°, 35°, 45°, 55°, 65°, 75°, and −45° relative to vertical (see Fig. 2). Using a differential conditioning paradigm, the 45° Gabor served as CS+ during acquisition, while the other six gratings became CS− stimuli. The −45° orientation was used as an additional condition with very high perceptual dissimilarity to the CS+. The US was a 98 dB (A) white noise, binaurally presented for 1000 ms via two speakers left and right behind the participant.

To evoke ssVEPs (Fig. 1B), each Gabor grating with a given orientation (i.e., each CS) was presented reversing its phase at either 14.167 Hz (10 participants) or 15 Hz (9 participants). We deliberately tested two distinct stimulation frequencies to increase external validity. Each CS presentation (trial) consisted of 71 phase reversals, yielding a CS trial-duration of 5012 ms at 14.167 Hz and 4734 ms at 15 Hz. During the acquisition phase, we extended each trial-duration for 14 additional phase reversals (corresponding to the duration of the 1 s US-presentation). EEG data recorded during US-presentation was excluded from subsequent analyses to avoid contamination of the ssVEP signal by the sound presentation. All gratings were created and all stimuli were presented in Psychophysics Toolbox (RRID:SCR_002881)^70,71^ running on MATLAB (RRID: SCR_001622).

In every learning phase each of the 8 CS orientations was presented 16 times (=16 trials of each CS - 128 trials in total per phase). The US was only presented during the acquisition phase. Here, US-presentation started 5012 ms (for 14.167 Hz) or 4734 ms (for 15 Hz) after the onset of every 45° Gabor grating (CS+, 100% reinforcement rate). In acquisition, 5 of the first 8 trials were always reinforced trials where the 45° CS+ co-terminated with the US. The remaining acquisition trials and all trials in habituation, extinction learning, and delayed recall were presented in a pseudorandom order in one of two predefined sequences, counterbalanced across participants (Supplementary Table S1 shows the exact sequences). As in previous studies^25^ these booster trials where introduced to assure learning of this rather difficult task, where one CS+ has to be identified among seven very similar CS−. The presentation order was restricted by the rule that no more than two consecutive presentations of the same CS orientation should occur within one conditioning phase. The inter-trial interval (ITI, offset to onset) was a black screen and ranged randomly between 4500 ms to 6500 ms, drawn from a uniform distribution. A white fixation cross was presented in the centre of the screen for the last 1500 ms of each ITI.

### EEG recording and pre-processing

We recorded EEG continuously from 64 active electrodes (Ag/AgCl, actiCAP, Brain Products) filled with electrolyte gel (Super-Visc 10%NaCl, EasyCap) with two 32-channel BrainAmp DC amplifiers (resolution 0.1µV, Brain Products) at a sampling rate of 1000 Hz with a high-pass filter at 0.08 Hz. Efforts were made to keep all impedances below 5 kΩ (manufacturer’s recommendation <25 kΩ). FCz was the recoding reference, AFz was ground. To control for vertical and horizontal eye-movement we recorded EOG with electrodes (Ø 4 mm, Ag/AgCl) placed close to the lateral canthus of each eye to record horizontal movements, as well as infra- and supra-orbital in line with the pupil of the right eye to capture vertical movements. To control for muscular blink activity we also recorded EMG of the orbicularis oculi muscle. EMG-electrodes (Ø 5 mm, Ag/AgCl) were placed over the left orbicularis oculi muscle underneath the eye lid, according to current guidelines^72^. A ground for EOG and EMG channels was placed on the forehead.

Pre-processing was accomplished off-line using BrainVision Analyzer 2 software. We used infinite impulse response (IIR), zero phase-shift, Butterworth filters to band-pass the non-segmented data with a high-pass filter with a cut-off frequency (3 dB point) of 0.5 Hz (roll-off: 48 dB/octave) and a low-pass at 40 Hz (3 dB point, 12 dB/octave). We also applied a 50 Hz notch filter (symmetrical, 5 Hz bandwidth, order 16). The data were then re-referenced to an average reference and the recording reference channel was reused as FCz, yielding 65 EEG channels. We then segmented the data from −600 to 5100 ms relative to the onset of each CS. The segments were down-sampled to 250 Hz and subjected to an ocular correction independent component analysis (ICA, as implemented in BrainVision Analyzer 2). After visual inspection of the resulting factors and factor topographies, factors related to horizontal and vertical eye movements, blinks, as well as strong cardiac or muscular artefacts were removed from the reconstructed data. Pre-processed EEG data were exported to MATLAB (RRID: SCR_001622). Trials with remaining artefacts were rejected based on visual inspection of butterfly-plots of each trial. For both experiment days, this left an average of 15.8 trials per condition (range: 13–16) for analysis. There were no significant differences between learning phases or conditions (all *F* < 3.34, all *p* > 0.070). Finally, we performed a scalp current source density (CSD) transform to the data^25,73^. The procedure diminishes volume conduction effects and delivers reference-free data. The CSD values (serving as estimates of cortical surface potentials) are represented on a sphere that approximates a cortical surface and thus serve as a mapping technique. In the present implementation^73^, the CSD is projected back onto the original electrode space to facilitate topographical mapping. All analyses were performed on CSD-transformed data, and CSD data are shown throughout the figures.

### ssVEP spectral analysis

The CSD values at each sensor were transformed into the frequency domain using a Fourier Transform of the average over 16 trials for each learning phase (habituation, acquisition, extinction learning, and day 2 delayed recall) and for each CS condition. For the 14.167 Hz stimulation, we used data from 988 to 5012 ms post stimulus onset (1006 sample points). For the 15 Hz stimulation, data from 972 to 4772 ms post stimulus onset was analysed (950 sample points). These time-series data were windowed with a cosine-square window (20 point rise/fall) and subjected to a discrete Fourier transform (MATLAB) with a frequency resolution of 0.2485 Hz at 14.167 Hz driving frequency and 0.2632 Hz at the 15 Hz driving frequency. Fourier coefficients were normalized by the length of the segment. The absolute value of the Fourier coefficients was extracted at the respective driving frequency. These power values were then converted to signal-to-noise ratios using the average power for the five frequency bins below and four frequency bins above the driving frequency. *Habituation corrected values:* To correct for interindividual variance in response strength and pre-experimental orientation bias, we converted occipital ssVEP power to a habituation ratio. For this purpose, we divided each subject’s mean occipital power for each CS orientation during acquisition, extinction, and day 2 recall trials by his respective mean power for the same orientation during habituation. This yielded an individual learning phase/habituation ratio for each specific CS. Here, values larger than 1 indicate an enhancement of responding to the specific CS relative to habituation (e.g., 1.5 would correspond to a 50% increase in ssVEP power relative to habituation).

### ssVEP single-trial analysis

We obtained estimates of single-trial ssVEP amplitude at the respective driving frequency (i.e., 14.167 & 15 Hz) by calculating a moving-window average for each trial and participant^74,75^. We used data starting 992 (for 14.167 Hz stimulation) and 972 ms (for 15 Hz) after stimulus onset to avoid contamination by the initial event-related brain potential (ERP). Each data segment was first baseline-corrected by subtracting the mean of a −600 to 0 ms pre-stimulus baseline period from each channel. A window with the length of four cycles of the respective driving frequency (14.167 and 15 Hz) was shifted across each detrended data segment in steps of one cycle (70.59 and 66.67 ms, for 14.167 and 15 Hz ssVEP, respectively), and the contents of the window were averaged with each step, resulting in averages containing four cycles of the respective ssVEP. A total of 53 averages were obtained for each trial and condition from each subject of the 14.167 Hz stimulation group (*N* = 10 subjects) and 52 averages for the 15 Hz group (*N* = 9), with the last window starting at 4492 ms for 14.167 Hz and at 4442 ms for 15 Hz. These 4-cycle averages of CSD values were transformed into the frequency domain using discrete Fourier transform (DFT). The Fourier coefficients were normalized by the length of the segment, and the power at the driving frequency was extracted. The data were then corrected for habituation by dividing each single-trial estimate by the mean over the 16 habituation trials within each participant, each CS, and each sensor. Finally, the data were smoothed with a 4-trial moving average.

### Skin conductance responses and EKG

Skin conductance was recorded with a 0.5 V constant voltage coupler at a sampling rate of 1000 Hz from two Ag/AgCl electrodes (Ø 10 mm), filled with 0.05 M NaCl paste (TD-246) and fixed on the thenar and hypothenar of the left hand. Skin conductance responses (SCR) to the CS were scored offline. A SCR was scored as the maximum onset-to-peak difference in conductance, with an onset occurring 1 to 4 s and a minimum amplitude of 0.02 µS. If more than one SCR met the criteria in a given trial their amplitudes were summed up. Responses not meeting these criteria were scored as zero. Scoring was blind to experimental conditions. SCRs were square-root transformed to normalize the distribution. Individual responses to each CS trial were transformed to standard z-scores within each participant using the mean and standard deviation computed over all CS responses from this participant.

For single-trial analysis of SCR data, we wanted to stay as comparable as possible to the single-trial analysis of ssVEP power. We took the square-root transformed responses from all CS trials. Within each subject we then subtracted the habituation average (16 trials) from each CS response. A division by the habituation average (as with ssVEP) was not possible, as some participants had a SCR habituation average of zero. Before statistical analysis, this habituation corrected SCR single-trial data were then smoothed within each participant with a 4-trial moving average.

Bipolar electrocardiogram (EKG) was recorded with the active electrodes (8 mm, Ag/AgCl) were placed on the left shinbone and under the right clavicle; ground was placed on the right shinbone. EKG data are not reported here.

### Ratings of CS valence and arousal, and US expectancy

The valence and arousal ratings were collected repeatedly (before and after each learning block, paper-pencil, to relieve participants’ eyes) for each Gabor grating, using the Self-Assessment Manikin^76^, a 9-point graphically presented and verbally anchored scale. Starting after acquisition, participants were also asked to rate to what extent they expected an US to occur with each particular CS (US expectancy rating). Ratings ranged from −5 (certainly no US), over 0 (uncertain) to 5 (certainly a US).

### Procedure

The main study consisted of two consecutive experiment days, starting either at 10:00 am, 2:00 pm, or 5:30 pm. Upon arrival at the lab on day 1, EEG and other physiology sensors (SCR, EOG, EMG, ECG, and blood pressure cuff) were attached and participants were seated in a dimly lit, sound-attenuated and electrically shielded experimental chamber. Day 1 included habituation, acquisition, and extinction learning as described above in “*Experimental design, fear conditioning stimuli and procedure”*. Prior to habituation participants were informed that they will see a series of flickering gratings at the centre of the monitor. They were also reminded to remain as still as possible and comfortably fixate the centre of the screen during the whole computer task. Prior to acquisition, participants were informed that a loud noise will be presented in combination with one of the gratings. However, they were not instructed as to which specific grating was going to predict the noise. In extinction learning (day 1) and delayed recall (day 2) the participants were not informed that no US would be presented. They were merely asked to remember the task instructions they were given earlier. The learning phases were interspaced with short rest periods (1 min), ratings of CS (after acquisition also US) valence and arousal as well as US expectancy. We also recorded resting-EEG, tonic SCR and ECG, and conducted a blood pressure measurement between learning phases. Here, participants were instructed to avoid any movement (except blinking) and to fixate the centre of the screen for 1.5 minutes.

### Statistical analysis

For all analyses, except single-trial analyses, we used (8 × 4) repeated measures ANOVA with the factors CS orientation (15, 25, 35, 45, 55, 65, 75 and −45 degrees) and a factor corresponding to the learning phase (habituation, acquisition, extinction, and day 2). These were followed up by ANOVAs for each learning phase (or rating time point) separately. To test the hypotheses of generalization vs. lateral inhibition, we then subjected the respective values to signed *F*-contrast tests. We used the weights reported by McTeague *et al*.^25^, where generalization was modelled as a quadratic trend with the weights: −3, 0.5, 1.5, 2, 1.5, 0.5, and −3 across means ordered by orientation (15° to 75°). Lateral inhibition was modelled as a ‘Mexican hat’ (difference of Gaussians) with the weights: 0.5, −1, −2, 5, −2, −1, and 0.5. Effect sizes reported are the *r*^2^_contrast_ values^77^. The fit of the competing models was compared using a Bayesian information criterion^41^. The BIC for a model *M1* was then approximated as BIC’^43^ from the *r*_contrast_ effect sizes as:

$${\rm{BIC}}{\mbox{'}}_{{\rm{M1}}}={\rm{n}}\,\mathrm{ln}(1\mbox{--}{r}_{{\rm{contrast}}\,{\rm{M}}1}^{2})+{\rm{k}}\,\mathrm{ln}({\rm{n}}),$$

where *n* is the number of subjects, and *k* = number of model parameters (set as *k* = 7 = number of weights for both models). We report the difference (ΔBIC) = BIC’ for model 1 minus BIC’ for the model 2 (the hypothesized model). ΔBIC values >6 suggest *strong*, and ΔBIC >12 suggest *very strong* evidence to prefer model 2 over model 1^42^.

Depending on the measure under analysis, the learning phase factor was defined differently. For uncorrected signal-to-noise ratios of ssVEP occipital power at the driving frequency, the factor included the 4 learning phases (i.e., habituation, acquisition, extinction learning, and day 2 delayed recall). For habituation-corrected ssVEP power, the factor included only 3 levels (acquisition, extinction learning, and day 2 delayed recall), because the habituation correction rendered values for habituation to be equal 1 in every case. For skin conductance responses, we analysed day 1 data separately averaged over all trials of habituation and acquisition. To demonstrate extinction, we compared the last 4 trials of acquisition with the last four trials of extinction learning. As a return-of-fear was only expected for the early portion of day 2 trials, we compared SCR data from the first four trials on day 2 to the last 4 trials of extinction learning on day 1. For subjective ratings the factor time point reflected the learning phase: for arousal and valence there were 5 rating time points (after habituation, after acquisition, after extinction, as well as before and after day 2 extinction trials); for US expectancy there were only 4 (after acquisition, after extinction, as well as before and after day 2 extinction trials). US expectancy has *N* = 18 due to missing ratings from one participant. In case of violations of the sphericity assumption, a Greenhouse-Geisser correction was applied.

For single-trial analyses of occipital ssVEP power and SCR we computed the *F*-contrasts for a ‘Mexican hat’ and a quadratic fit (custom MATLAB code based on^25^) for each trial. We addressed multiple comparisons by calculating permutation *F*-distributions from data where CS orientations and learning phases were randomly shuffled within participants with a total of 4000 *F*-tests entering each distribution. The 0.95 quantile of this distribution served as the critical *F*-value for each dependent variable.

We computed the scalp distribution of the ‘Mexican hat’ vs. quadratic fits (Figs. 2C and 4A) by applying the same contrast weights to habituation-corrected power estimates of each of the 65 scalp electrodes for the average over acquisition, extinction, and day 2 trials (implemented with custom MATLAB code based on^25^ and visualized in the EMEGS software^78^). Significance level for all analyses was set at 0.05 and all tests were two-tailed.

## Supplementary information

Supplementary Figures & Tables.

## Data Availability

The data that support the findings of this study are available on request from the corresponding author (M.I.A.).

## Code availability

Custom MATLAB scripts used for analyses are available from the corresponding author (M.I.A.) upon request.
