# Supplementary material for: Visuocortical tuning to a threat-related feature persists after extinction and consolidation of conditioned fear
Source: Sci Rep. 2020 Mar 3;10:3926. doi: 10.1038/s41598-020-60597-z (PMC7054355; doi:10.1038/s41598-020-60597-z)
Supplement: Supplementary file 1 — Supplementary Figures & Tables. [file 41598_2020_60597_MOESM1_ESM.pdf]

**Supplementary Figures & Tables.**

**Title:** Visuocortical tuning to a threat-related feature persists after extinction and consolidation of conditioned fear.

**Authors:** Martin I. Antov, Elena Plog, Philipp Bierwirth, Andreas Keil, and Ursula Stockhorst

***Note.*** To assure the comparability of our figures with previous work using this specific fear conditioning task (McTeague et al. *Nat. Commun.* **6**, 7823 [2015]) we have plotted values as *Mean* ± 1 *SEM* in Figures 2, 4, and 5 of the manuscript. The Supplementary Figures S1, S2, and S6-S8 included here, show the same data with a combination of dot plots (representing individual values of every subject that entered the respective statistical analysis) and boxplots. This should help with transparency and allow the reader to judge the full variance and distribution of the raw data.

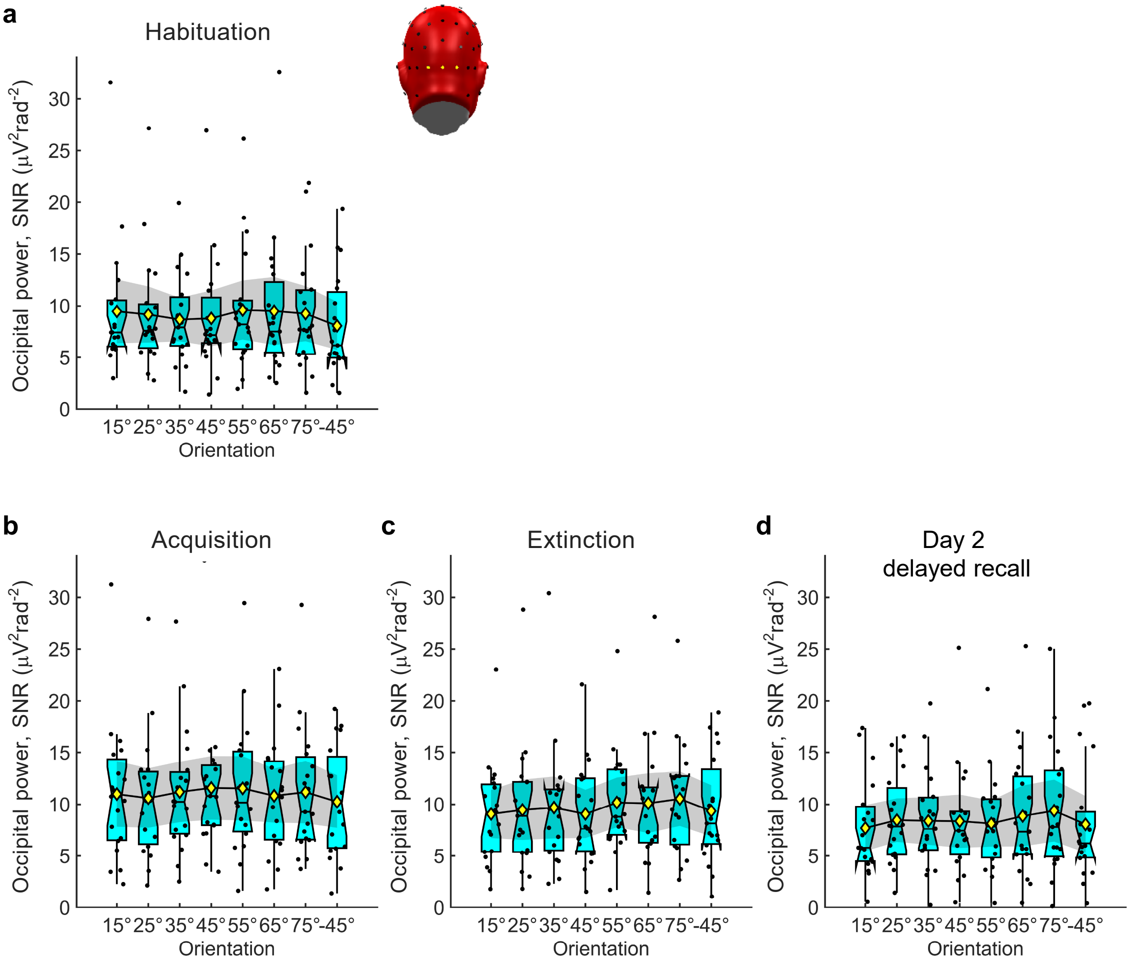


**Suppl. Fig. S1 | Occipital cortical responses during the different phases of conditioning.** Changes in visual electrocortical activity for the learning phases **(a)** *habituation*, **(b)** *acquisition*, **(c)** *extinction*, and **(d)** *day 2 delayed recall*, shown for each CS-orientation (on the x-axes). In all sub-plots **(a-d)** the data show ssVEP spectral power current source density (CSD, Laplacian space), averaged across 3 occipital midline sensor locations (O1, Oz, O2). All values are signal to noise-ratios (SNR), i.e., the power at the driving frequency was divided by the average power for the five frequency bins below and four frequency bins above the driving frequency (as the noise estimate). The insert to the right from subplot **(a)** shows a view of the back of the electrode array used, the sensor locations used for averaging are highlighted in yellow. These data are shown in the manuscript in Fig. 2b with $M \pm 1$ *SEM*.

Each ***black dot*** shows the SNR of a single subject ($N = 19$). For the ***boxplots***: the cyan ***boxes*** are drawn between the $25^{th}$ and $75^{th}$ percentile, the ***horizontal line*** marks the median. The boxplot ***whiskers*** extend above and below the box to the most extreme data points that are within a distance to the box equal to 1.5 times the interquartile range (Tukey boxplot). The narrowing of the boxes displays ***notches*** at median ± 1.58 x interquartile range / ($\sqrt{N}$). ***Yellow diamonds*** connected with a solid black line show the means ($N = 19$) and the ***gray shaded area*** around the means shows the 95% confidence interval of the mean.

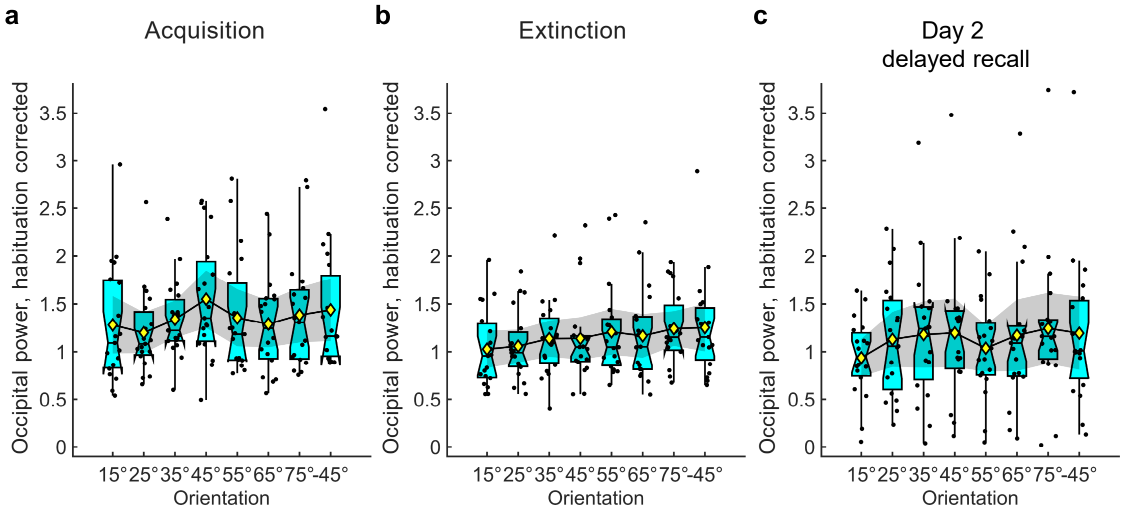


**Suppl. Fig. S2 | The same data as in Suppl. Fig. 1 after habituation correction.** Each subject's occipital power for each CS-orientation during acquisition, extinction, and day 2 delayed recall was normalized through division by the corresponding orientation's mean power during habituation: **(a)** *acquisition*, **(b)** *extinction*, and **(c)** *day 2 delayed recall.* These data are shown in the manuscript in Fig. 2b with $M \pm 1$ *SEM*.

Each ***black dot*** shows the SNR of a single subject ($N = 19$). For the ***boxplots***: the cyan ***boxes*** are drawn between the 25th and 75th percentile, the ***horizontal line*** marks the median. The boxplot ***whiskers*** extend above and below the box to the most extreme data points that are within a distance to the box equal to 1.5 times the interquartile range (Tukey boxplot). The narrowing of the boxes displays ***notches*** at median ± 1.58 x interquartile range / ($\sqrt{N}$). ***Yellow diamonds*** connected with a solid black line show the means ($N = 19$) and the ***gray shaded area*** around the means shows the 95% confidence interval of the mean.

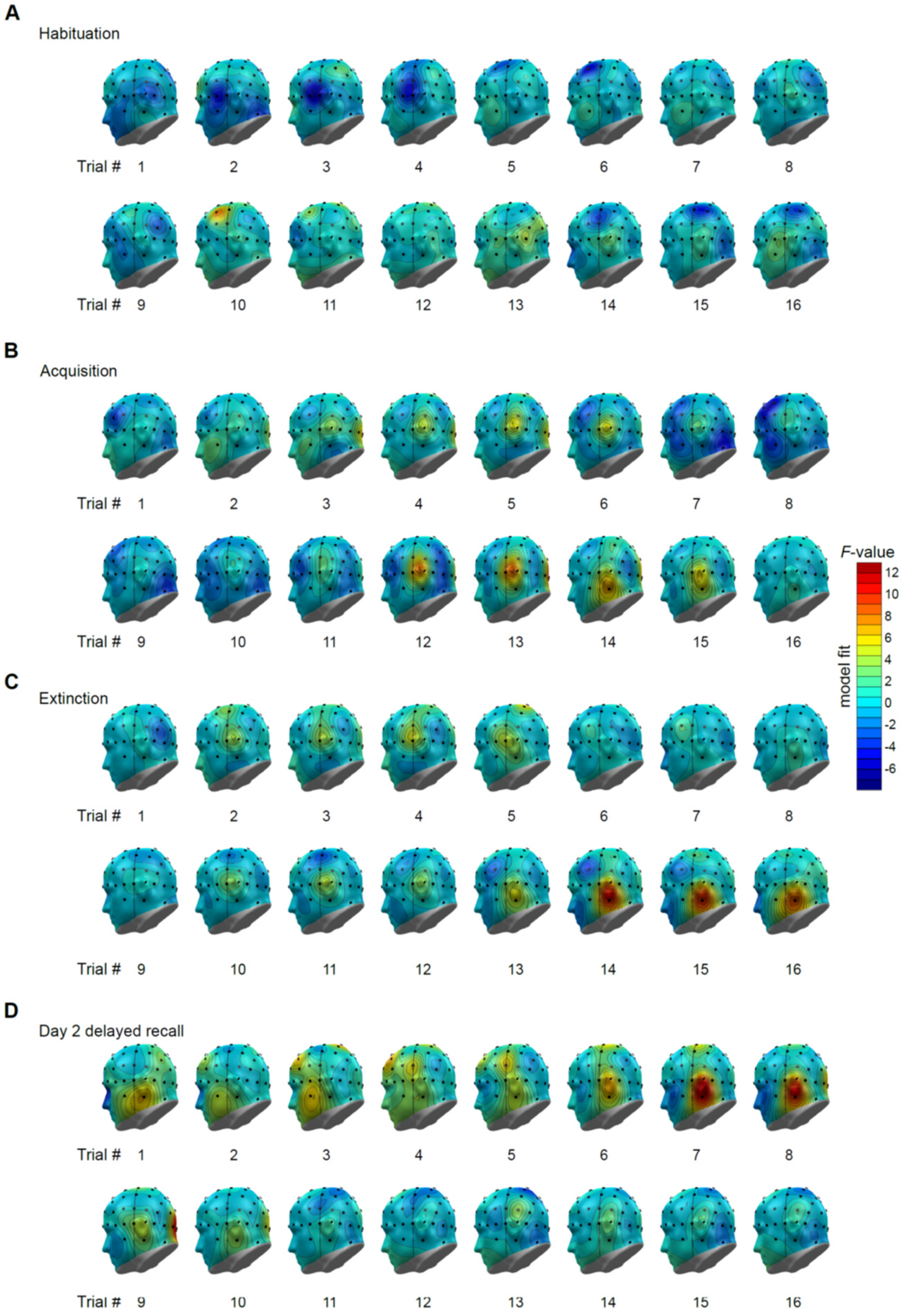


**Suppl. Fig. S3 | Topographical distributions of 'Mexican hat' statistical fits over single trial data, left view of the scalp.** Color maps show *F*-values, black dots the EEG-electrode positions. **(A)** *Habituation*, **(B)** *acquisition*, **(C)** *extinction*, and **(D)** *day 2 delayed recall.* Parts of these (trials 5-12 from day 2) are shown in the manuscript Fig. 4A.

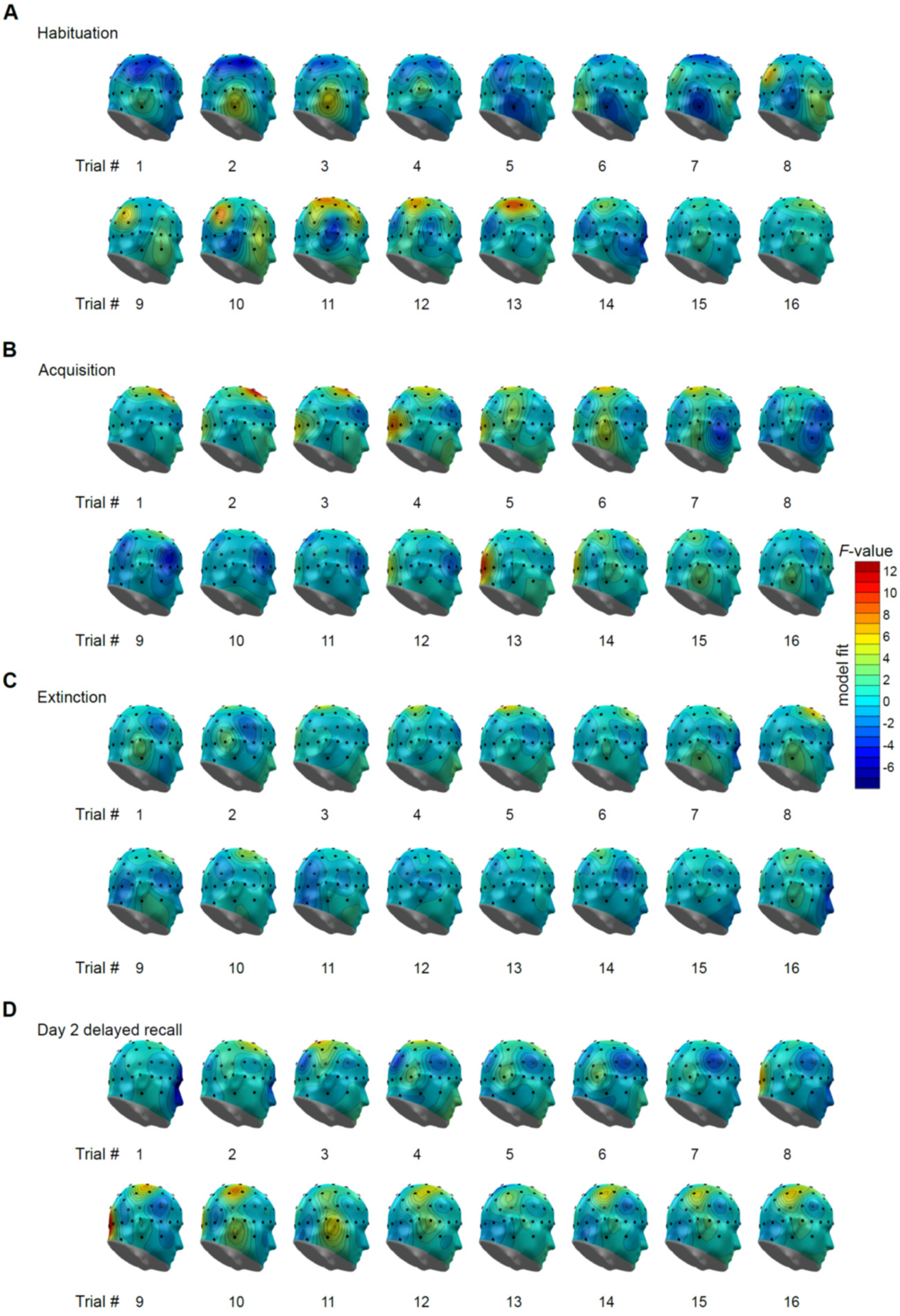


**Suppl. Fig. S4 | Topographical distributions of 'Mexican hat' statistical fits over single trial data, right view of the scalp.** Color maps show *F*-values, black dots the EEG-electrode positions. **(A)** *Habituation*, **(B)** *acquisition*, **(C)** *extinction*, and **(D)** *day 2 delayed recall.* Parts of these (trials 5-12 from day 2) are shown in the manuscript Fig. 4A.

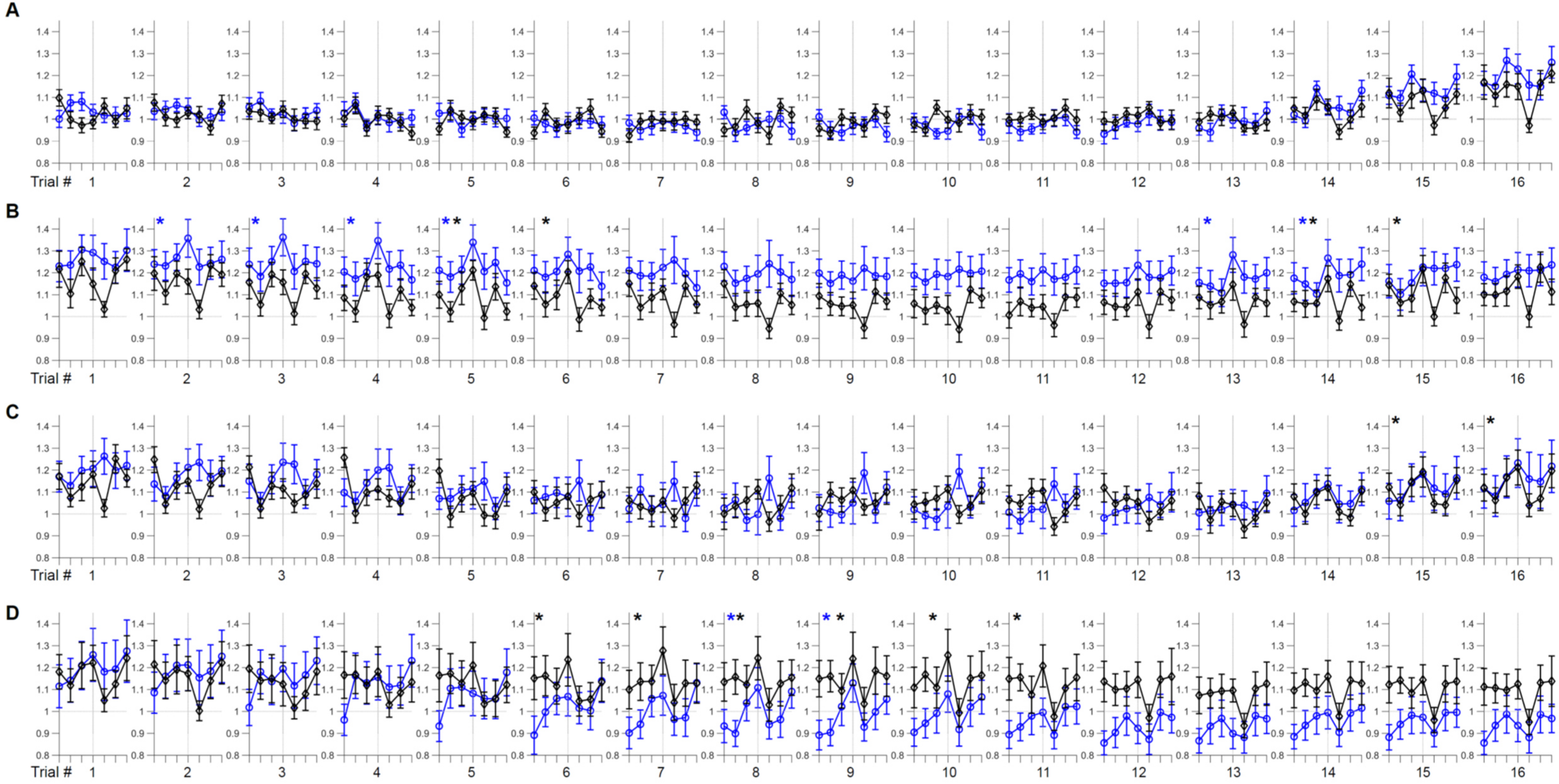

**Suppl. Fig. S5 | Alternative depiction of single-trial data for a priori occipital and exploratory bilateral temporo-occipital regions.** Changes in visual electrocortical activity for the learning phases **(A)** *habituation*, **(B)** *acquisition*, **(C)** *extinction*, and **(D)** *day 2 delayed recall*. Each subplot shows data from one trial, CS-orientation is on the x-axes (left to right: 15°, 25°, 45°, 55°, 65°, 75°), vertical grey dotted lines mark the CS+ (45°). Data shown are the same as in the contour plots in manuscript Fig. 3A and Fig. 4B, here as line plots with error estimates. In all sub-plots **(A-D)** the ***blue line*** shows data averaged across the 3 a priori occipital midline sensor locations (O1, Oz, O2); the ***black line*** shows data averaged across the 4 sensors of the exploratory bilateral temporo-occipital region (TP8, TP10, TP7, and TP9). As in manuscript Fig. 3A and 4B, all values (y-axes) are changes in single-trial power estimates at the driving frequency, relative to habituation, i.e. power for each data point divided by the average of the 16 habituation single-trial estimates at the respective CS orientation. Asterisks at the upper left of a subplot denote a substantial 'Mexican hat' fit (contrast *F*-value > 4.25). Error bars show ± 1 *SEM*.

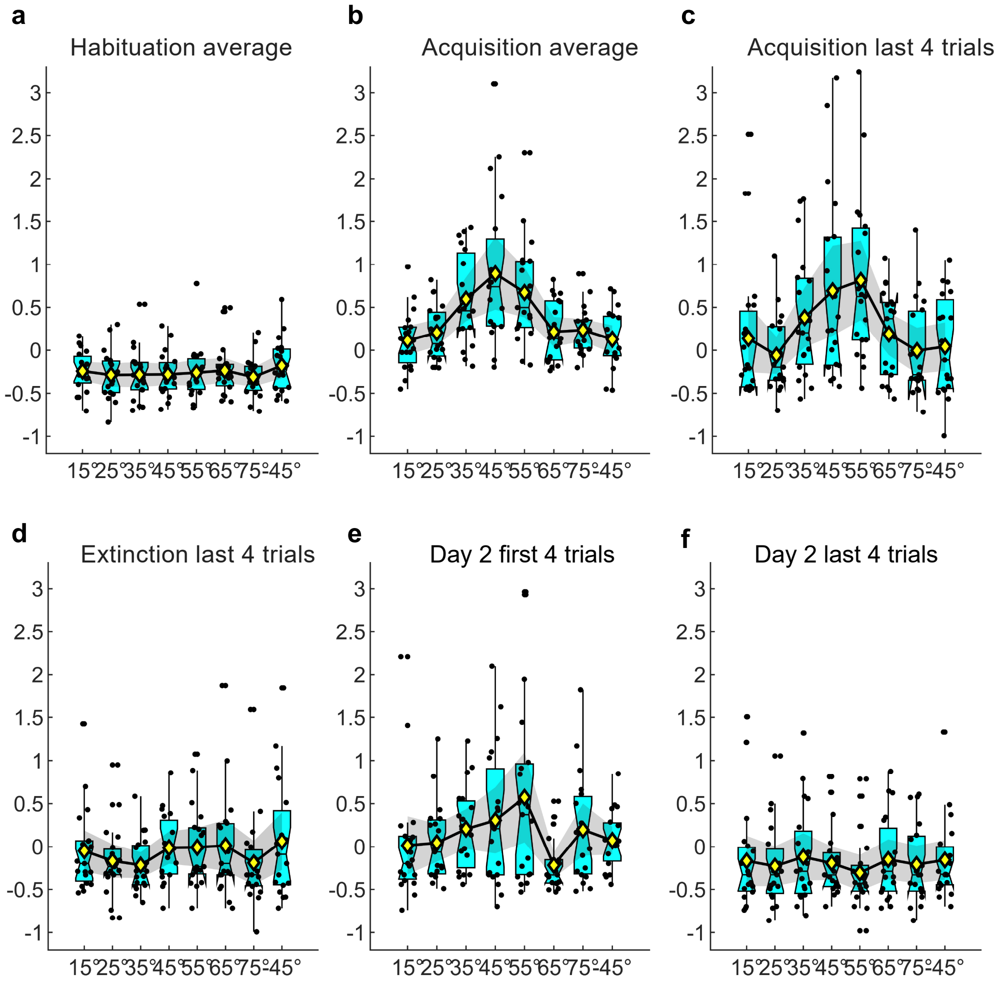


**Suppl. Fig. S6 | Skin conductance responses during the different phases of fear conditioning. (a)** *Habituation average,* **(b)** *acquisition average*, **(c)** *acquisition last 4 trials,* **(d)** *extinction last 4 trials*, **(e)** *first 4 trials*, and **(f)** *last 4 trials* of *delayed recall on day 2***.** The data (*N* = 19) are individual z-scores standardized on the mean and SD of all responses in the experiment. These data are shown in the manuscript in Fig. 5a and b with *M* ± 1 *SEM*.

Each ***black dot*** shows the z-score of a single participant (*N* = 19, averaged over 16 trials for day 1, and over 8 trials for day 2). For the ***boxplots***: the cyan ***boxes*** are drawn between the 25$^{th}$ and 75$^{th}$ percentile, the ***horizontal line*** marks the median. The boxplot ***whiskers*** extend above and below the box to the most extreme data points that are within a distance to the box equal to 1.5 times the interquartile range (Tukey boxplot). The narrowing of the boxes displays ***notches*** at median ± 1.58 x interquartile range / (√ *N*). ***Yellow diamonds*** connected with a solid black line show the means (*N = 19*) and the ***gray shaded area*** around the means shows the 95% confidence interval of the mean.

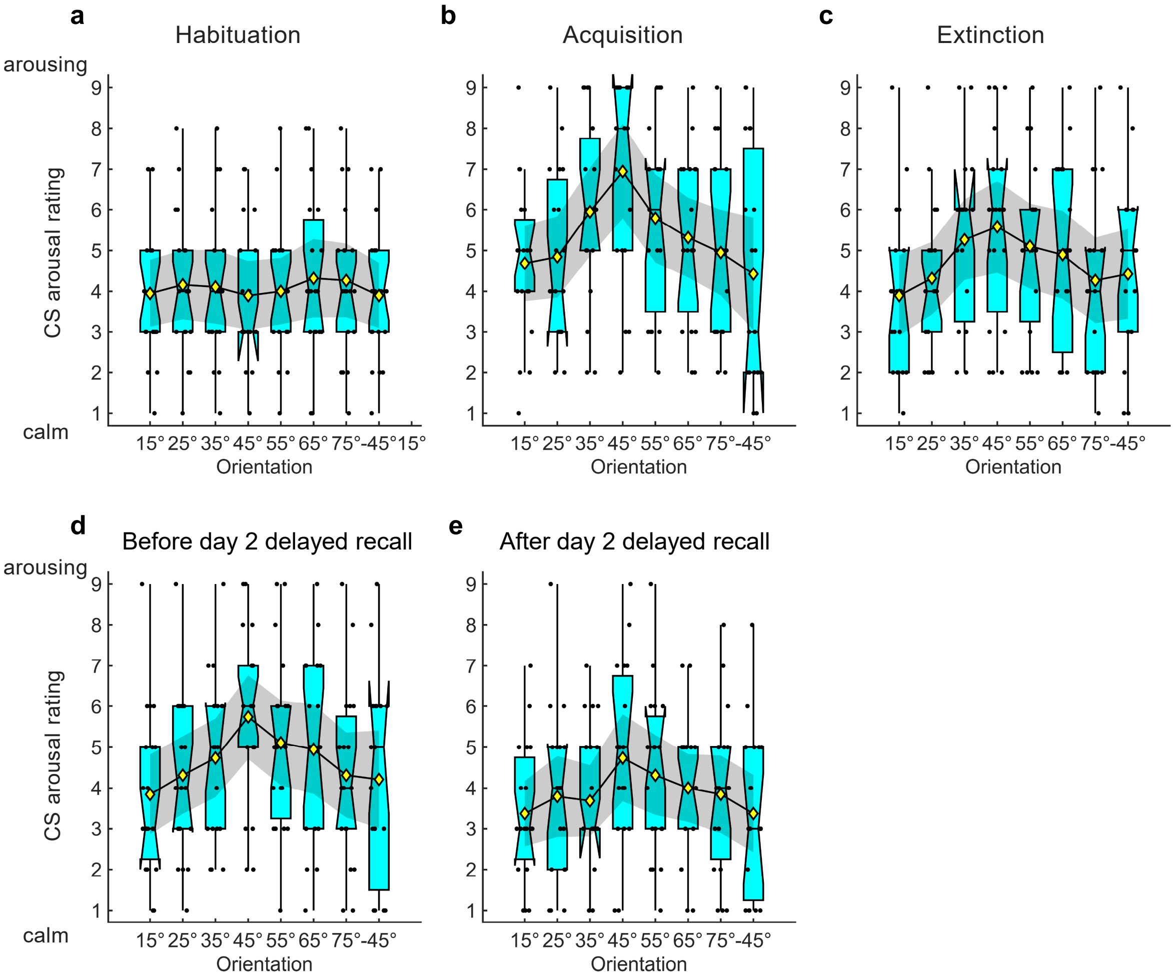


**Suppl. Fig. S7 | Subjective self-report changes in CS arousal ratings during the different phases of fear conditioning.** Values for each CS-orientation from all $N$ = 19 subjects, as rated on day 1 **(a)** *after habituation*, **(b)** *after acquisition*, and **(c)** after extinction, as well as **(d)** *before,* and **(e)** *after delayed recall on day 2*. These data are shown in the manuscript in Fig. 5a and b with $M$ ± 1 *SEM*.

Each ***black dot*** shows the rating of a single participant ($N$ = 19). For the ***boxplots***: the cyan ***boxes*** are drawn between the 25$^{th}$ and 75$^{th}$ percentile, the ***horizontal line*** marks the median. The boxplot ***whiskers*** extend above and below the box to the most extreme data points that are within a distance to the box equal to 1.5 times the interquartile range (Tukey boxplot). The narrowing of the boxes displays ***notches*** at median ± 1.58 x interquartile range / (√ $N$). ***Yellow diamonds*** connected with a solid black line show the means ($N$ = *19*) and the ***gray shaded area*** around the means shows the 95% confidence interval of the mean.

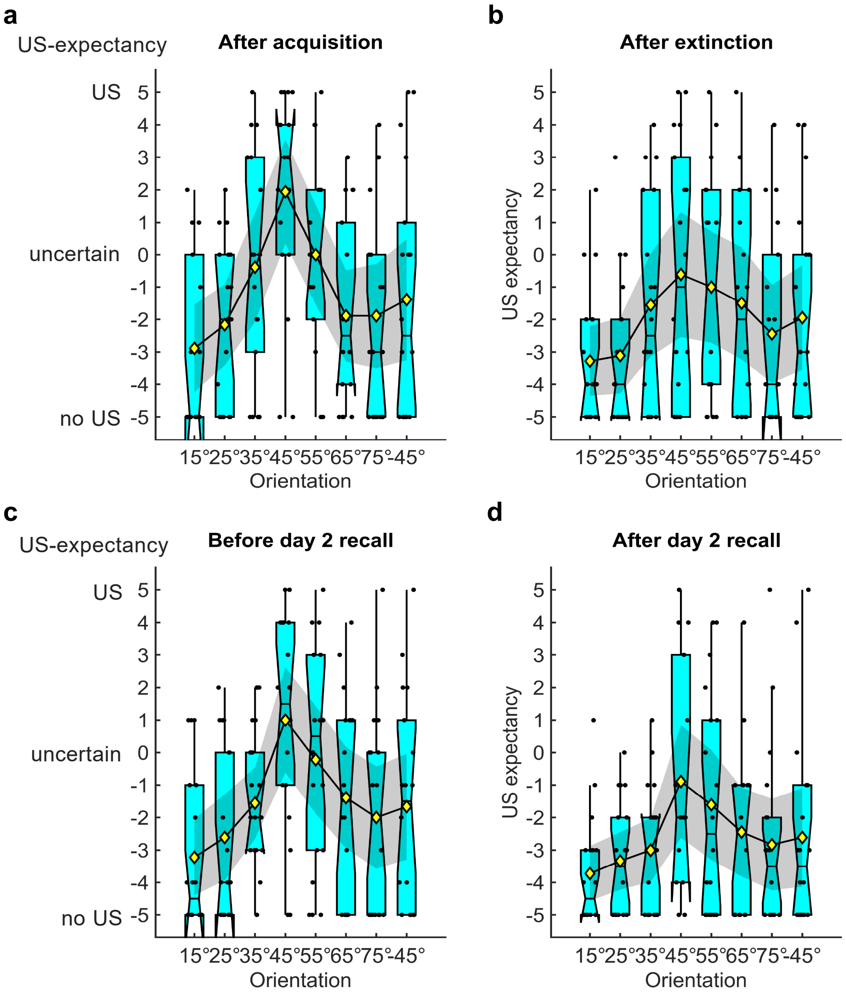


**Suppl. Fig. S8 | Subjective self-report changes in US-expectancy ratings during the different phases of fear conditioning.** Values for each CS-orientation from $N$ = 18 (one participant did not provide US-expectancy ratings), as rated on day 1 **(a)** *after acquisition*, and **(b)** after extinction, as well as **(c)** *before,* and **(d)** *after delayed recall on day 2*. These data are shown in the manuscript in Fig. 5c and d with $M \pm 1$ *SEM*.

Each ***black dot*** shows the US-expectancy rating of a single participant ($N$ = 18). For the ***boxplots***: the cyan ***boxes*** are drawn between the 25th and 75th percentile, the ***horizontal line*** marks the median. The boxplot ***whiskers*** extend above and below the box to the most extreme data points that are within a distance to the box equal to 1.5 times the interquartile range (Tukey boxplot). The narrowing of the boxes displays ***notches*** at median ± 1.58 x interquartile range / ($\sqrt{N}$). ***Yellow diamonds*** connected with a solid black line show the means ($N$ = *18*) and the ***gray shaded area*** around the means shows the 95% confidence interval of the mean.

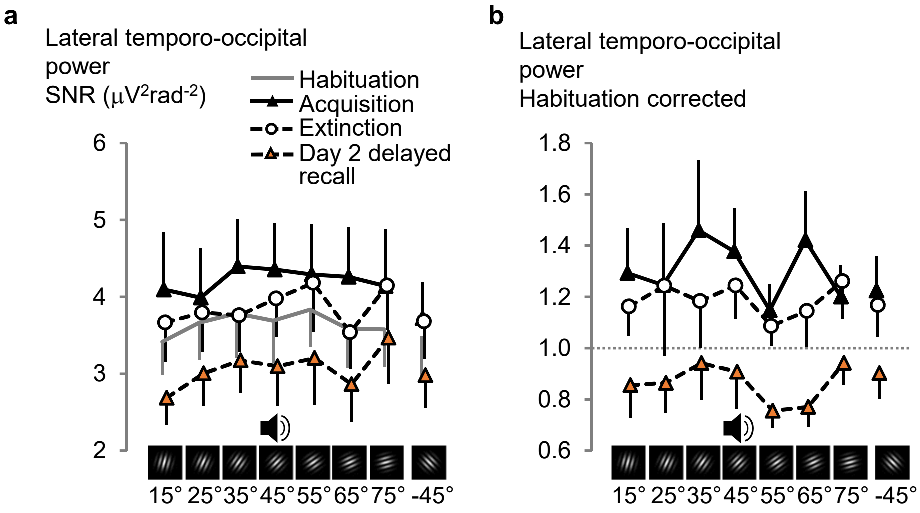


**Suppl. Fig. S9 | As in manuscript Fig. 2A, 2B, for completeness: Lateral temporo-occipital cortical responses averaged over 16 trials for each phase of conditioning. (a)** Changes in the grand average ($N$ = 19) of visual electrocortical activity for each learning phase (habituation, acquisition, extinction, and day 2 delayed recall) and for each CS orientation. Regional means of the ssVEP spectral power current source density (CSD, Laplacian space), averaged across the bilateral temporo-occipital region pooled from the explorative 4-sensor cluster (TP8, TP10, TP7, and TP9). Values are signal-to-noise ratios (SNR), i.e., the power at the driving frequency was divided by the average power for the five frequency bins below and four frequency bins above the driving frequency (as the noise estimate). **(b)** The same data after habituation correction for acquisition, extinction, and day 2 delayed recall.

**Suppl. Table 1. Trial order of stimuli during the experiment in squence 1 and sequence 2**

**HABITUATION**

| Sequence 1 | | | Sequence 2 | | |
|---|---|---|---|---|---|
| Trial # | Repeat # | CS orientation | Trial # | Repeat # | CS orientation |
| 1 | 1 | 55 | 1 | 1 | -45 |
| 2 | 1 | 15 | 2 | 1 | **45** |
| 3 | 1 | -45 | 3 | 1 | 25 |
| 4 | 1 | 25 | 4 | 2 | -45 |
| 5 | 2 | 15 | 5 | 1 | 35 |
| 6 | 2 | 55 | 6 | 1 | 65 |
| 7 | 1 | 65 | 7 | 1 | 15 |
| 8 | 2 | 65 | 8 | 2 | 35 |
| 9 | 1 | **45** | 9 | 3 | -45 |
| 10 | 1 | 75 | 10 | 3 | 35 |
| 11 | 3 | 15 | 11 | 2 | 25 |
| 12 | 2 | 75 | 12 | 2 | **45** |
| 13 | 1 | 35 | 13 | 1 | 55 |
| 14 | 2 | 25 | 14 | 1 | 75 |
| 15 | 3 | 75 | 15 | 2 | 55 |
| 16 | 2 | 35 | 16 | 2 | 75 |
| 17 | 2 | **45** | 17 | 2 | 65 |
| 18 | 3 | 65 | 18 | 3 | **45** |
| 19 | 2 | -45 | 19 | 2 | 15 |
| 20 | 4 | 65 | 20 | 3 | 65 |
| 21 | 3 | 25 | 21 | 3 | 75 |
| 22 | 3 | -45 | 22 | 3 | 15 |
| 23 | 3 | 35 | 23 | 4 | 35 |
| 24 | 3 | 55 | 24 | 4 | **45** |
| 25 | 4 | -45 | 25 | 3 | 55 |
| 26 | 4 | 15 | 26 | 4 | 55 |
| 27 | 4 | 35 | 27 | 4 | 75 |
| 28 | 4 | 55 | 28 | 4 | 65 |
| 29 | 4 | 75 | 29 | 4 | -45 |
| 30 | 3 | **45** | 30 | 3 | 25 |
| 31 | 4 | **45** | 31 | 4 | 15 |
| 32 | 4 | 25 | 32 | 4 | 25 |
| 33 | 5 | -45 | 33 | 5 | 65 |
| 34 | 5 | 25 | 34 | 6 | 65 |
| 35 | 5 | 55 | 35 | 5 | 55 |
| 36 | 5 | **45** | 36 | 5 | 35 |
| 37 | 5 | 15 | 37 | 7 | 65 |
| 38 | 6 | 55 | 38 | 5 | -45 |
| 39 | 6 | 25 | 39 | 8 | 65 |
| 40 | 7 | 25 | 40 | 5 | **45** |
| 41 | 5 | 65 | 41 | 6 | 55 |
| 42 | 6 | -45 | 42 | 5 | 15 |
| 43 | 6 | **45** | 43 | 7 | 55 |
| 44 | 6 | 65 | 44 | 5 | 75 |
| 45 | 7 | 65 | 45 | 9 | 65 |
| 46 | 7 | -45 | 46 | 6 | 75 |
| 47 | 5 | 75 | 47 | 6 | 15 |
| 48 | 7 | 55 | 48 | 5 | 25 |
| 49 | 5 | 35 | 49 | 6 | **45** |
| 50 | 6 | 15 | 50 | 7 | **45** |
| 51 | 6 | 35 | 51 | 8 | 55 |
| 52 | 8 | 25 | 52 | 6 | 35 |
| 53 | 8 | -45 | 53 | 6 | 25 |
| 54 | 6 | 75 | 54 | 9 | 55 |
| 55 | 7 | 75 | 55 | 7 | 35 |
| 56 | 8 | 55 | 56 | 6 | -45 |
| 57 | 8 | 65 | 57 | 7 | 25 |
| 58 | 7 | 35 | 58 | 7 | 15 |
| 59 | 7 | **45** | 59 | 7 | 75 |
| 60 | 7 | 15 | 60 | 8 | **45** |
| 61 | 8 | 35 | 61 | 8 | 35 |
| 62 | 9 | 55 | 62 | 7 | -45 |
| 63 | 8 | **45** | 63 | 8 | 25 |
| 64 | 9 | 65 | 64 | 8 | -45 |
| 65 | 9 | 35 | 65 | 9 | **45** |
| 66 | 8 | 75 | 66 | 8 | 75 |
| 67 | 9 | **45** | 67 | 9 | -45 |
| 68 | 9 | -45 | 68 | 9 | 25 |
| 69 | 10 | 35 | 69 | 9 | 35 |
| 70 | 10 | 65 | 70 | 10 | 55 |
| 71 | 9 | 75 | 71 | 10 | 35 |
| 72 | 10 | 55 | 72 | 11 | 35 |
| 73 | 8 | 15 | 73 | 9 | 75 |
| 74 | 11 | 35 | 74 | 10 | 25 |
| 75 | 11 | 65 | 75 | 10 | **45** |
| 76 | 11 | 55 | 76 | 10 | -45 |
| 77 | 10 | **45** | 77 | 8 | 15 |
| 78 | 11 | **45** | 78 | 9 | 15 |
| 79 | 9 | 25 | 79 | 10 | 75 |
| 80 | 12 | 65 | 80 | 11 | **45** |
| 81 | 10 | 25 | 81 | 12 | 35 |
| 82 | 9 | 15 | 82 | 10 | 65 |
| 83 | 10 | -45 | 83 | 11 | 65 |
| 84 | 10 | 75 | 84 | 12 | **45** |
| 85 | 11 | 25 | 85 | 10 | 15 |
| 86 | 10 | 15 | 86 | 11 | 25 |
| 87 | 12 | 25 | 87 | 11 | 15 |
| 88 | 12 | **45** | 88 | 11 | 75 |
| 89 | 11 | 75 | 89 | 12 | 75 |
| 90 | 11 | -45 | 90 | 12 | 65 |
| 91 | 12 | 55 | 91 | 12 | 15 |
| 92 | 11 | 15 | 92 | 11 | -45 |
| 93 | 12 | 15 | 93 | 11 | 55 |
| 94 | 12 | -45 | 94 | 12 | 25 |
| 95 | 12 | 35 | 95 | 12 | -45 |
| 96 | 12 | 75 | 96 | 12 | 55 |
| 97 | 13 | 75 | 97 | 13 | -45 |
| 98 | 13 | 65 | 98 | 13 | **45** |
| 99 | 14 | 65 | 99 | 13 | 25 |
| 100 | 13 | **45** | 100 | 13 | 35 |
| 101 | 13 | 15 | 101 | 13 | 65 |
| 102 | 13 | -45 | 102 | 13 | 75 |
| 103 | 13 | 35 | 103 | 13 | 55 |
| 104 | 13 | 25 | 104 | 14 | 65 |
| 105 | 14 | 25 | 105 | 14 | **45** |
| 106 | 15 | 65 | 106 | 14 | 35 |
| 107 | 14 | 15 | 107 | 14 | 25 |
| 108 | 13 | 55 | 108 | 13 | 15 |
| 109 | 14 | 35 | 109 | 15 | **45** |
| 110 | 15 | 25 | 110 | 15 | 35 |
| 111 | 15 | 15 | 111 | 14 | -45 |
| 112 | 14 | -45 | 112 | 14 | 75 |
| 113 | 14 | **45** | 113 | 16 | 35 |
| 114 | 14 | 75 | 114 | 15 | 65 |
| 115 | 15 | -45 | 115 | 14 | 55 |
| 116 | 14 | 55 | 116 | 15 | 25 |
| 117 | 15 | 75 | 117 | 15 | -45 |
| 118 | 15 | **45** | 118 | 16 | **45** |
| 119 | 16 | 75 | 119 | 16 | 25 |
| 120 | 15 | 55 | 120 | 15 | 55 |
| 121 | 16 | **45** | 121 | 14 | 15 |
| 122 | 16 | 65 | 122 | 16 | 55 |
| 123 | 16 | 15 | 123 | 16 | -45 |
| 124 | 15 | 35 | 124 | 15 | 15 |
| 125 | 16 | 25 | 125 | 16 | 65 |
| 126 | 16 | -45 | 126 | 15 | 75 |
| 127 | 16 | 35 | 127 | 16 | 15 |
| 128 | 16 | 55 | 128 | 16 | 75 |

**ACQUISITION**

| Sequence 1 | | | Sequence 2 | | |
|---|---|---|---|---|---|
| Trial # | Repeat # | CS orientation | Trial # | Repeat # | CS orientation |
| 1 | 1 | **45** | 1 | 1 | **45** |
| 2 | 2 | **45** | 2 | 2 | **45** |
| 3 | 1 | 25 | 3 | 1 | 25 |
| 4 | 3 | **45** | 4 | 3 | **45** |
| 5 | 1 | 65 | 5 | 1 | 65 |
| 6 | 1 | -45 | 6 | 1 | -45 |
| 7 | 4 | **45** | 7 | 4 | **45** |
| 8 | 5 | **45** | 8 | 5 | **45** |
| 9 | 1 | 35 | 9 | 2 | 65 |
| 10 | 1 | 55 | 10 | 1 | 35 |
| 11 | 2 | -45 | 11 | 1 | 55 |
| 12 | 2 | 25 | 12 | 6 | **45** |
| 13 | 1 | 15 | 13 | 1 | 15 |
| 14 | 1 | 75 | 14 | 2 | 15 |
| 15 | 3 | 25 | 15 | 2 | -45 |
| 16 | 2 | 15 | 16 | 2 | 25 |
| 17 | 6 | **45** | 17 | 3 | 65 |
| 18 | 2 | 65 | 18 | 3 | 15 |
| 19 | 3 | 65 | 19 | 4 | 65 |
| 20 | 2 | 75 | 20 | 1 | 75 |
| 21 | 7 | **45** | 21 | 2 | 75 |
| 22 | 2 | 55 | 22 | 2 | 35 |
| 23 | 3 | -45 | 23 | 3 | -45 |
| 24 | 3 | 15 | 24 | 4 | 15 |
| 25 | 4 | 25 | 25 | 7 | **45** |
| 26 | 4 | 15 | 26 | 5 | 65 |
| 27 | 3 | 75 | 27 | 3 | 75 |
| 28 | 4 | 65 | 28 | 4 | -45 |
| 29 | 3 | 55 | 29 | 5 | -45 |
| 30 | 4 | -45 | 30 | 6 | 65 |
| 31 | 2 | 35 | 31 | 3 | 25 |
| 32 | 4 | 75 | 32 | 3 | 35 |
| 33 | 5 | 65 | 33 | 2 | 55 |
| 34 | 5 | -45 | 34 | 3 | 55 |
| 35 | 3 | 35 | 35 | 4 | 75 |
| 36 | 4 | 55 | 36 | 4 | 55 |
| 37 | 6 | 65 | 37 | 8 | **45** |
| 38 | 8 | **45** | 38 | 4 | 25 |
| 39 | 5 | 25 | 39 | 5 | 25 |
| 40 | 4 | 35 | 40 | 5 | 15 |
| 41 | 5 | 35 | 41 | 4 | 35 |
| 42 | 5 | 15 | 42 | 6 | -45 |
| 43 | 6 | 35 | 43 | 5 | 35 |
| 44 | 9 | **45** | 44 | 6 | 15 |
| 45 | 5 | 55 | 45 | 6 | 35 |
| 46 | 6 | 25 | 46 | 9 | **45** |
| 47 | 6 | -45 | 47 | 6 | 25 |
| 48 | 7 | 25 | 48 | 7 | 35 |
| 49 | 7 | 35 | 49 | 5 | 55 |
| 50 | 8 | 25 | 50 | 8 | 35 |
| 51 | 6 | 55 | 51 | 7 | 65 |
| 52 | 10 | **45** | 52 | 6 | 55 |
| 53 | 6 | 15 | 53 | 5 | 75 |
| 54 | 7 | 55 | 54 | 8 | 65 |
| 55 | 8 | 35 | 55 | 7 | 55 |
| 56 | 7 | 65 | 56 | 7 | 25 |
| 57 | 5 | 75 | 57 | 8 | 55 |
| 58 | 9 | 25 | 58 | 9 | 65 |
| 59 | 10 | 25 | 59 | 7 | -45 |
| 60 | 8 | 55 | 60 | 10 | **45** |
| 61 | 6 | 75 | 61 | 7 | 15 |
| 62 | 8 | 65 | 62 | 8 | 25 |
| 63 | 9 | 55 | 63 | 9 | 55 |
| 64 | 7 | 15 | 64 | 9 | 25 |
| 65 | 10 | 55 | 65 | 10 | 25 |
| 66 | 9 | 65 | 66 | 8 | 15 |
| 67 | 8 | 15 | 67 | 6 | 75 |
| 68 | 7 | -45 | 68 | 10 | 55 |
| 69 | 11 | **45** | 69 | 11 | 55 |
| 70 | 9 | 15 | 70 | 8 | -45 |
| 71 | 11 | 55 | 71 | 7 | 75 |
| 72 | 12 | 55 | 72 | 12 | 55 |
| 73 | 10 | 65 | 73 | 8 | 75 |
| 74 | 7 | 75 | 74 | 11 | **45** |
| 75 | 11 | 25 | 75 | 9 | 15 |
| 76 | 9 | 35 | 76 | 12 | **45** |
| 77 | 8 | -45 | 77 | 9 | -45 |
| 78 | 9 | -45 | 78 | 9 | 35 |
| 79 | 13 | 55 | 79 | 13 | 55 |
| 80 | 8 | 75 | 80 | 11 | 25 |
| 81 | 11 | 65 | 81 | 12 | 25 |
| 82 | 10 | 15 | 82 | 10 | 35 |
| 83 | 9 | 75 | 83 | 10 | 15 |
| 84 | 12 | 25 | 84 | 13 | 25 |
| 85 | 12 | **45** | 85 | 9 | 75 |
| 86 | 10 | -45 | 86 | 10 | 75 |
| 87 | 14 | 55 | 87 | 10 | 65 |
| 88 | 13 | **45** | 88 | 11 | 75 |
| 89 | 10 | 75 | 89 | 11 | 35 |
| 90 | 15 | 55 | 90 | 13 | **45** |
| 91 | 12 | 65 | 91 | 10 | -45 |
| 92 | 13 | 25 | 92 | 11 | 15 |
| 93 | 11 | 75 | 93 | 11 | -45 |
| 94 | 11 | 15 | 94 | 14 | 55 |
| 95 | 11 | -45 | 95 | 12 | 15 |
| 96 | 10 | 35 | 96 | 11 | 65 |
| 97 | 11 | 35 | 97 | 15 | 55 |
| 98 | 12 | 15 | 98 | 12 | 65 |
| 99 | 12 | 75 | 99 | 13 | 15 |
| 100 | 13 | 65 | 100 | 14 | **45** |
| 101 | 14 | 65 | 101 | 12 | 75 |
| 102 | 14 | **45** | 102 | 14 | 25 |
| 103 | 12 | -45 | 103 | 13 | 65 |
| 104 | 12 | 35 | 104 | 13 | 75 |
| 105 | 14 | 25 | 105 | 14 | 15 |
| 106 | 13 | -45 | 106 | 12 | -45 |
| 107 | 15 | 65 | 107 | 15 | 15 |
| 108 | 13 | 75 | 108 | 13 | -45 |
| 109 | 14 | -45 | 109 | 12 | 35 |
| 110 | 14 | 75 | 110 | 14 | -45 |
| 111 | 13 | 15 | 111 | 14 | 65 |
| 112 | 14 | 15 | 112 | 15 | -45 |
| 113 | 13 | 35 | 113 | 15 | 25 |
| 114 | 15 | -45 | 114 | 16 | 15 |
| 115 | 15 | 25 | 115 | 13 | 35 |
| 116 | 15 | 15 | 116 | 15 | **45** |
| 117 | 16 | 25 | 117 | 14 | 35 |
| 118 | 14 | 35 | 118 | 14 | 75 |
| 119 | 15 | **45** | 119 | 15 | 65 |
| 120 | 15 | 75 | 120 | 15 | 75 |
| 121 | 16 | -45 | 121 | 16 | 65 |
| 122 | 16 | 75 | 122 | 15 | 35 |
| 123 | 16 | **45** | 123 | 16 | 25 |
| 124 | 15 | 35 | 124 | 16 | 35 |
| 125 | 16 | 15 | 125 | 16 | **45** |
| 126 | 16 | 35 | 126 | 16 | 75 |
| 127 | 16 | 55 | 127 | 16 | -45 |
| 128 | 16 | 65 | 128 | 16 | 55 |

**EXTINCTION**

| Sequence 1 | | | Sequence 2 | | |
|---|---|---|---|---|---|
| Trial # | Repeat # | CS orientation | Trial # | Repeat # | CS orientation |
| 1 | 1 | 55 | 1 | 1 | -45 |
| 2 | 1 | 35 | 2 | 1 | 65 |
| 3 | 1 | -45 | 3 | 1 | 25 |
| 4 | 1 | 25 | 4 | 1 | 55 |
| 5 | 2 | 35 | 5 | 1 | 35 |
| 6 | 1 | 15 | 6 | 1 | 15 |
| 7 | 1 | 65 | 7 | 2 | -45 |
| 8 | 1 | **45** | 8 | 2 | 35 |
| 9 | 2 | 55 | 9 | 2 | 65 |
| 10 | 1 | 75 | 10 | 1 | 75 |
| 11 | 2 | **45** | 11 | 2 | 75 |
| 12 | 2 | 75 | 12 | 2 | 55 |
| 13 | 3 | 55 | 13 | 2 | 15 |
| 14 | 2 | -45 | 14 | 3 | 15 |
| 15 | 3 | 75 | 15 | 2 | 25 |
| 16 | 3 | **45** | 16 | 3 | 65 |
| 17 | 3 | -45 | 17 | 3 | 35 |
| 18 | 2 | 15 | 18 | 1 | **45** |
| 19 | 4 | -45 | 19 | 2 | **45** |
| 20 | 3 | 35 | 20 | 3 | -45 |
| 21 | 4 | 55 | 21 | 3 | **45** |
| 22 | 3 | 15 | 22 | 3 | 55 |
| 23 | 2 | 65 | 23 | 3 | 75 |
| 24 | 2 | 25 | 24 | 4 | 65 |
| 25 | 3 | 25 | 25 | 4 | -45 |
| 26 | 4 | 35 | 26 | 4 | 35 |
| 27 | 4 | 25 | 27 | 4 | **45** |
| 28 | 4 | 15 | 28 | 4 | 15 |
| 29 | 4 | **45** | 29 | 4 | 75 |
| 30 | 3 | 65 | 30 | 3 | 25 |
| 31 | 4 | 65 | 31 | 4 | 25 |
| 32 | 4 | 75 | 32 | 4 | 55 |
| 33 | 5 | 25 | 33 | 5 | 35 |
| 34 | 5 | **45** | 34 | 5 | **45** |
| 35 | 6 | **45** | 35 | 5 | 55 |
| 36 | 5 | 75 | 36 | 6 | 55 |
| 37 | 5 | 55 | 37 | 6 | 35 |
| 38 | 5 | 35 | 38 | 5 | 15 |
| 39 | 5 | 15 | 39 | 7 | 55 |
| 40 | 5 | -45 | 40 | 5 | -45 |
| 41 | 6 | 55 | 41 | 5 | 75 |
| 42 | 6 | 35 | 42 | 5 | 25 |
| 43 | 6 | -45 | 43 | 5 | 65 |
| 44 | 6 | 25 | 44 | 6 | -45 |
| 45 | 5 | 65 | 45 | 6 | 15 |
| 46 | 7 | -45 | 46 | 7 | -45 |
| 47 | 6 | 65 | 47 | 6 | 65 |
| 48 | 7 | **45** | 48 | 7 | 65 |
| 49 | 7 | 35 | 49 | 6 | 25 |
| 50 | 6 | 75 | 50 | 8 | 55 |
| 51 | 7 | 25 | 51 | 7 | 25 |
| 52 | 8 | 35 | 52 | 8 | -45 |
| 53 | 7 | 75 | 53 | 7 | 15 |
| 54 | 6 | 15 | 54 | 7 | 35 |
| 55 | 8 | 75 | 55 | 8 | 35 |
| 56 | 8 | **45** | 56 | 6 | 75 |
| 57 | 7 | 65 | 57 | 6 | **45** |
| 58 | 8 | 65 | 58 | 7 | 75 |
| 59 | 7 | 55 | 59 | 8 | 15 |
| 60 | 7 | 15 | 60 | 7 | **45** |
| 61 | 8 | 25 | 61 | 8 | 65 |
| 62 | 8 | -45 | 62 | 8 | **45** |
| 63 | 8 | 15 | 63 | 8 | 25 |
| 64 | 8 | 55 | 64 | 8 | 75 |
| 65 | 9 | 75 | 65 | 9 | 55 |
| 66 | 9 | 35 | 66 | 9 | 25 |
| 67 | 9 | -45 | 67 | 9 | -45 |
| 68 | 9 | 15 | 68 | 9 | 35 |
| 69 | 10 | 15 | 69 | 10 | -45 |
| 70 | 9 | 55 | 70 | 9 | **45** |
| 71 | 10 | -45 | 71 | 10 | **45** |
| 72 | 10 | 75 | 72 | 9 | 15 |
| 73 | 9 | **45** | 73 | 11 | -45 |
| 74 | 9 | 25 | 74 | 9 | 75 |
| 75 | 11 | 15 | 75 | 10 | 15 |
| 76 | 10 | 25 | 76 | 10 | 75 |
| 77 | 11 | 75 | 77 | 10 | 25 |
| 78 | 11 | -45 | 78 | 11 | 25 |
| 79 | 12 | 15 | 79 | 10 | 35 |
| 80 | 11 | 25 | 80 | 9 | 65 |
| 81 | 9 | 65 | 81 | 10 | 65 |
| 82 | 12 | 25 | 82 | 10 | 55 |
| 83 | 10 | **45** | 83 | 11 | 75 |
| 84 | 11 | **45** | 84 | 11 | 65 |
| 85 | 10 | 55 | 85 | 11 | 15 |
| 86 | 10 | 65 | 86 | 11 | **45** |
| 87 | 10 | 35 | 87 | 11 | 55 |
| 88 | 12 | -45 | 88 | 12 | 65 |
| 89 | 11 | 55 | 89 | 11 | 35 |
| 90 | 12 | 75 | 90 | 12 | 75 |
| 91 | 11 | 65 | 91 | 12 | 25 |
| 92 | 11 | 35 | 92 | 12 | 55 |
| 93 | 12 | 65 | 93 | 12 | 15 |
| 94 | 12 | **45** | 94 | 12 | 35 |
| 95 | 12 | 55 | 95 | 12 | **45** |
| 96 | 12 | 35 | 96 | 12 | -45 |
| 97 | 13 | 15 | 97 | 13 | 35 |
| 98 | 13 | **45** | 98 | 13 | -45 |
| 99 | 13 | 75 | 99 | 14 | 35 |
| 100 | 13 | 35 | 100 | 13 | 55 |
| 101 | 14 | 15 | 101 | 13 | 75 |
| 102 | 14 | **45** | 102 | 13 | 65 |
| 103 | 14 | 35 | 103 | 14 | 75 |
| 104 | 13 | 65 | 104 | 14 | 55 |
| 105 | 13 | 55 | 105 | 13 | **45** |
| 106 | 14 | 75 | 106 | 15 | 35 |
| 107 | 15 | 75 | 107 | 14 | **45** |
| 108 | 13 | -45 | 108 | 15 | 55 |
| 109 | 13 | 25 | 109 | 13 | 25 |
| 110 | 15 | 35 | 110 | 15 | 75 |
| 111 | 15 | 15 | 111 | 14 | 25 |
| 112 | 16 | 35 | 112 | 13 | 15 |
| 113 | 14 | 55 | 113 | 14 | 65 |
| 114 | 16 | 75 | 114 | 15 | 65 |
| 115 | 14 | -45 | 115 | 15 | 25 |
| 116 | 14 | 65 | 116 | 16 | 55 |
| 117 | 15 | 65 | 117 | 16 | 75 |
| 118 | 15 | **45** | 118 | 14 | -45 |
| 119 | 15 | -45 | 119 | 15 | -45 |
| 120 | 16 | 65 | 120 | 14 | 15 |
| 121 | 14 | 25 | 121 | 16 | 65 |
| 122 | 15 | 25 | 122 | 16 | -45 |
| 123 | 15 | 55 | 123 | 15 | 15 |
| 124 | 16 | 15 | 124 | 16 | 25 |
| 125 | 16 | **45** | 125 | 15 | **45** |
| 126 | 16 | 55 | 126 | 16 | 35 |
| 127 | 16 | 25 | 127 | 16 | **45** |
| 128 | 16 | -45 | 128 | 16 | 15 |

**DAY 2 DELAYED RECALL**

| Sequence 1 | | | Sequence 2 | | |
|---|---|---|---|---|---|
| Trial # | Repeat # | CS orientation | Trial # | Repeat # | CS orientation |
| 1 | 1 | -45 | 1 | 1 | 75 |
| 2 | 1 | 15 | 2 | 1 | 55 |
| 3 | 1 | 35 | 3 | 2 | 55 |
| 4 | 1 | **45** | 4 | 1 | 25 |
| 5 | 1 | 75 | 5 | 2 | 75 |
| 6 | 2 | **45** | 6 | 3 | 55 |
| 7 | 1 | 25 | 7 | 1 | -45 |
| 8 | 1 | 55 | 8 | 1 | 15 |
| 9 | 2 | 15 | 9 | 1 | 35 |
| 10 | 3 | **45** | 10 | 2 | 25 |
| 11 | 3 | 15 | 11 | 3 | 25 |
| 12 | 2 | 35 | 12 | 1 | **45** |
| 13 | 3 | 35 | 13 | 3 | 75 |
| 14 | 1 | 65 | 14 | 2 | **45** |
| 15 | 2 | 25 | 15 | 4 | 75 |
| 16 | 2 | -45 | 16 | 2 | 35 |
| 17 | 2 | 55 | 17 | 1 | 65 |
| 18 | 2 | 75 | 18 | 2 | 65 |
| 19 | 4 | 35 | 19 | 4 | 55 |
| 20 | 3 | 25 | 20 | 3 | 65 |
| 21 | 3 | 75 | 21 | 3 | 35 |
| 22 | 2 | 65 | 22 | 2 | 15 |
| 23 | 4 | 25 | 23 | 2 | -45 |
| 24 | 4 | **45** | 24 | 3 | **45** |
| 25 | 3 | 55 | 25 | 4 | 35 |
| 26 | 3 | -45 | 26 | 3 | 15 |
| 27 | 4 | 15 | 27 | 3 | -45 |
| 28 | 4 | 55 | 28 | 4 | 65 |
| 29 | 3 | 65 | 29 | 4 | **45** |
| 30 | 4 | 75 | 30 | 4 | 25 |
| 31 | 4 | -45 | 31 | 4 | 15 |
| 32 | 4 | 65 | 32 | 4 | -45 |
| 33 | 5 | **45** | 33 | 5 | -45 |
| 34 | 5 | 25 | 34 | 5 | 65 |
| 35 | 5 | 35 | 35 | 5 | **45** |
| 36 | 6 | 25 | 36 | 5 | 75 |
| 37 | 5 | 55 | 37 | 5 | 55 |
| 38 | 6 | 55 | 38 | 6 | -45 |
| 39 | 6 | **45** | 39 | 6 | 75 |
| 40 | 7 | 55 | 40 | 6 | 55 |
| 41 | 5 | 75 | 41 | 5 | 25 |
| 42 | 6 | 35 | 42 | 6 | 25 |
| 43 | 5 | 15 | 43 | 5 | 15 |
| 44 | 8 | 55 | 44 | 5 | 35 |
| 45 | 5 | -45 | 45 | 7 | 25 |
| 46 | 7 | **45** | 46 | 6 | 65 |
| 47 | 6 | 15 | 47 | 6 | 35 |
| 48 | 6 | -45 | 48 | 7 | 75 |
| 49 | 6 | 75 | 49 | 7 | 65 |
| 50 | 7 | 15 | 50 | 7 | 55 |
| 51 | 7 | 75 | 51 | 6 | **45** |
| 52 | 5 | 65 | 52 | 7 | 35 |
| 53 | 6 | 65 | 53 | 7 | **45** |
| 54 | 7 | 25 | 54 | 8 | 35 |
| 55 | 8 | 75 | 55 | 8 | **45** |
| 56 | 8 | 15 | 56 | 8 | 75 |
| 57 | 8 | **45** | 57 | 6 | 15 |
| 58 | 7 | 35 | 58 | 8 | 25 |
| 59 | 7 | 65 | 59 | 8 | 65 |
| 60 | 8 | 65 | 60 | 7 | -45 |
| 61 | 7 | -45 | 61 | 7 | 15 |
| 62 | 8 | -45 | 62 | 8 | 15 |
| 63 | 8 | 25 | 63 | 8 | 55 |
| 64 | 8 | 35 | 64 | 8 | -45 |
| 65 | 9 | -45 | 65 | 9 | 15 |
| 66 | 9 | **45** | 66 | 9 | 35 |
| 67 | 9 | 25 | 67 | 10 | 35 |
| 68 | 9 | 65 | 68 | 9 | 25 |
| 69 | 9 | 75 | 69 | 9 | 55 |
| 70 | 9 | 35 | 70 | 9 | -45 |
| 71 | 10 | 35 | 71 | 10 | 55 |
| 72 | 10 | -45 | 72 | 9 | **45** |
| 73 | 10 | 25 | 73 | 9 | 65 |
| 74 | 10 | 65 | 74 | 11 | 55 |
| 75 | 11 | 25 | 75 | 10 | -45 |
| 76 | 10 | **45** | 76 | 10 | **45** |
| 77 | 11 | 35 | 77 | 10 | 25 |
| 78 | 10 | 75 | 78 | 10 | 65 |
| 79 | 9 | 55 | 79 | 11 | 65 |
| 80 | 10 | 55 | 80 | 9 | 75 |
| 81 | 9 | 15 | 81 | 11 | -45 |
| 82 | 12 | 25 | 82 | 11 | **45** |
| 83 | 11 | 65 | 83 | 10 | 75 |
| 84 | 11 | 75 | 84 | 11 | 35 |
| 85 | 10 | 15 | 85 | 10 | 15 |
| 86 | 11 | -45 | 86 | 12 | 35 |
| 87 | 11 | 55 | 87 | 11 | 25 |
| 88 | 12 | 75 | 88 | 11 | 15 |
| 89 | 11 | **45** | 89 | 11 | 75 |
| 90 | 12 | **45** | 90 | 12 | 65 |
| 91 | 11 | 15 | 91 | 12 | 15 |
| 92 | 12 | 35 | 92 | 12 | 55 |
| 93 | 12 | 65 | 93 | 12 | -45 |
| 94 | 12 | 15 | 94 | 12 | 75 |
| 95 | 12 | 55 | 95 | 12 | 25 |
| 96 | 12 | -45 | 96 | 12 | **45** |
| 97 | 13 | 15 | 97 | 13 | 15 |
| 98 | 13 | 35 | 98 | 13 | 35 |
| 99 | 13 | 55 | 99 | 13 | 65 |
| 100 | 14 | 15 | 100 | 13 | 75 |
| 101 | 14 | 35 | 101 | 14 | 35 |
| 102 | 13 | **45** | 102 | 14 | 15 |
| 103 | 15 | 15 | 103 | 13 | **45** |
| 104 | 16 | 15 | 104 | 14 | 75 |
| 105 | 13 | 75 | 105 | 15 | 75 |
| 106 | 14 | 55 | 106 | 13 | 55 |
| 107 | 15 | 35 | 107 | 14 | **45** |
| 108 | 14 | **45** | 108 | 14 | 55 |
| 109 | 14 | 75 | 109 | 16 | 75 |
| 110 | 13 | 65 | 110 | 13 | 25 |
| 111 | 13 | 25 | 111 | 15 | 35 |
| 112 | 14 | 65 | 112 | 15 | 55 |
| 113 | 15 | 75 | 113 | 14 | 25 |
| 114 | 15 | 65 | 114 | 13 | -45 |
| 115 | 15 | **45** | 115 | 14 | -45 |
| 116 | 16 | 35 | 116 | 15 | 15 |
| 117 | 16 | 65 | 117 | 14 | 65 |
| 118 | 13 | -45 | 118 | 16 | 15 |
| 119 | 16 | 75 | 119 | 16 | 55 |
| 120 | 14 | -45 | 120 | 15 | -45 |
| 121 | 15 | 55 | 121 | 15 | 65 |
| 122 | 15 | -45 | 122 | 16 | 65 |
| 123 | 14 | 25 | 123 | 16 | 35 |
| 124 | 15 | 25 | 124 | 15 | 25 |
| 125 | 16 | **45** | 125 | 15 | **45** |
| 126 | 16 | -45 | 126 | 16 | **45** |
| 127 | 16 | 55 | 127 | 16 | -45 |
| 128 | 16 | 25 | 128 | 16 | 25 |
